# Anti‐PD‐1 Nanobody‐Armored MSLN CAR‐T Therapy for Malignant Mesothelioma: Preclinical and Clinical Studies

**DOI:** 10.1002/advs.202508754

**Published:** 2025-10-24

**Authors:** Yan Sun, Haochen Yang, Qing Xu, Xingya Li, Jinxing Lou, Jianchun Duan, Jiachen Xu, Zhuqing Liu, Yong Xia, Zhicai Lin, Linlin Li, Dan Sun, Jiaguo Li, Tao Liu, Jun Guo, Wenfeng Xu, Weimin Zhu, Yi Liu, Boyang Sun, Jia Zhong, Lijie Rong, Qijun Qian, Chenqi Xu, Jie Wang

**Affiliations:** ^1^ School of Medicine Shanghai Mengchao Cancer Hospital Shanghai University Shanghai 200444 China; ^2^ State Key Laboratory of Molecular Biology Shanghai Science Research Center CAS Center for Excellence in Molecular Cell Science Shanghai Institute of Biochemistry and Cell Biology Chinese Academy of Sciences University of Chinese Academy of Sciences Shanghai 200031 China; ^3^ Department of Medical Oncology Cancer Hospital Chinese Academy of Medical Sciences Beijing 100021 China; ^4^ Department of Oncology Shanghai Tenth People's Hospital Tongji University Cancer Center Tongji University Shanghai 200072 China; ^5^ Department of Oncology Department of Gynecological Oncology the First Affiliated Hospital of Zhengzhou University Zhengzhou 450052 China; ^6^ Shanghai Cell Therapy Group Co. Ltd Shanghai 201805 China

**Keywords:** CAR‐T, first‐in‐human study, malignant mesothelioma, NAC‐T

## Abstract

Malignant mesothelioma (MM) is an aggressive and currently incurable cancer with limited therapeutic options. Due to the high expression of mesothelin in this cancer, anti‐PD‐1 nanobody‐armored mesothelin‐targeting CAR‐T (NAC‐T) cells are developed. Based on the enhanced anti‐tumor activity observed in preclinical in vitro and in vivo studies, a first‐in‐human clinical trial is initiated. Eleven patients with malignant mesothelioma who have progressed after standard therapies receive intravenous infusions of 5–20 × 10^6^ per kg NAC‐T cells following lymphodepletion. The treatment is well tolerated, with no dose‐limiting toxicity observed. The overall response rate is 63.6%, including one complete response, and the disease control rate is 100%. The median progression‐free survival is 5.0 months, and the median overall survival is 25.6 months. Moreover, T cell receptor and single‐cell sequencing analyses in patients with varying responses revealed specific clonal expansion of T cell subtypes and enhanced reactivity to tumor‐associated antigens. These findings suggest that NAC‐T cell therapy represents a promising therapeutic strategy for patients with malignant mesothelioma.

## Graphical abstract

A novel therapy using engineered immune cells (NAC‐T cells) showed promise for refractory malignant mesothelioma. Based on the encouraging preclinical data, the first‐in‐human trial is initiated, demonstrating tolerable safety and promising anti‐tumor activity (ORR 63.6%, DCR 100%, including one CR). Further exploration reveals that NAC‐T works by directly killing tumor cells and boosting the surrounding immune response.

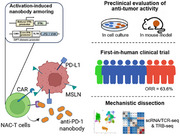

## 1 Introduction

Malignant mesothelioma (MM) is a highly aggressive malignancy that originates from the mesothelium lining the serous body cavities, such as the pleura (85%), peritoneum (15%), and other sites (<1%), and has limited treatment options. Both malignant pleural mesothelioma (MPM) and malignant peritoneal mesothelioma (MPeM) have poor prognoses, with a reported 1‐year overall survival (OS) rate below 50%.^[^ ^1^, ^2^ ^]^ The standard first‐line treatments for advanced malignant mesothelioma include pemetrexed‐based chemotherapy and immune checkpoint inhibitors (ICIs). For relapsed/recurrent malignant mesothelioma, second‐line treatment options remain limited, with an overall response rate (ORR) below 30% and a disease control rate (DCR) below 70%, as exhibited by clinical trials of mono‐ or combined therapies with ICIs such as nivolumab and ipilimumab,^[^ ^3^, ^4^ ^]^ highlighting the urgent need for the development of innovative therapeutic options.

Mesothelin (MSLN) has emerged as a promising target for cancer therapy because of its restricted expression in normal tissues and overexpression in various malignancies, including mesothelioma.^[^ ^5^ ^]^ Chimeric antigen receptor T cell (CAR‐T) therapy is an emerging immunotherapy that harnesses T cells to eliminate tumor cells expressing target antigens. Although favorable tolerability has been reported for MSLN‐targeted CAR‐T therapy, anti‐tumor activity remained limited in multiple clinical trials.^[^ ^6^, ^7^, ^8^ ^]^ A major challenge in improving efficacy is preventing CAR‐T cell exhaustion in the immunosuppressive tumor microenvironment (TME).^[^ ^9^, ^10^ ^]^ A recent clinical study showed that combinatorial treatment with pembrolizumab significantly improved the anti‐tumor activity of MSLN CAR‐T cells in patients with malignant mesothelioma,^[^ ^11^ ^]^ highlighting the potential benefit of modulating programmed death receptor‐1 (PD‐1) signaling in CAR‐T cells. However, pembrolizumab was initiated ≈6 weeks after CAR‐T cell infusion, which complicated the management of CAR‐T cell exhaustion during the later declining phase.

To counteract CAR‐T cell exhaustion promptly after their activation, several preclinical studies have engineered CAR‐T cells armored with self‐secreted anti‐PD‐1 antibody,^[^ ^12^, ^13^, ^14^, ^15^ ^]^ exhibiting enhanced proliferation and anti‐tumor activity, but still lacking clinical evidence. However, the amount of self‐secreted anti‐PD‐1 antibody is extremely low in the TME,^[^ ^12^, ^16^ ^]^ indicating the feasibility and necessity of boosting the productivity or facilitating penetration of the anti‐PD‐1 antibody. The variable domain of the heavy chain of the heavy‐chain‐only antibody (VHH antibody, also known as nanobody) has 1/10 of the size of the full‐length antibody, facilitating its production and intratumor distribution while maintaining comparable affinity. Therefore, nanobodies as armament have significant potential to enhance the clinical efficacy of CAR‐T cell therapy.

In our previous work, we developed an immune cell‐specific chimeric promoter for CAR‐T cells and demonstrated its preclinical utility in enhancing the production of anti‐PD‐1 antibody by engineered T cells.^[^ ^17^ ^]^ Based on these findings, we aimed to optimize PD‐1 blockade using nanobody technology to maximize therapeutic efficacy and facilitate clinical translation. Here, we developed MSLN‐targeting, nanobody‐armored CAR‐T (NAC‐T) cells that are equipped with an inducible anti‐PD‐1 nanobody payload, aiming to exploit the immunosuppressive TME through localized PD‐1 blockade, thereby boosting the anti‐tumor activity of CAR‐T cells. The safety and efficacy of these NAC‐T cells were subsequently evaluated in a first‐in‐human clinical study against MSLN‐expressing malignant mesothelioma.

## 2 Results

### 2.1 Alpaca‐Derived Anti‐PD‐1 Nanobody was Designed to Armor MSLN CAR‐T Cells

PD‐1 expression is significantly induced in exhausted tumor‐infiltrating T cells or CAR‐T cells, both in vitro and in vivo. To counteract PD‐1‐mediated T‐cell exhaustion, we designed CAR‐T cells that self‐secrete an anti‐PD‐1 antibody, utilizing a non‐viral *piggyBac* gene modification system as we previously reported.^[^ ^17^, ^18^, ^19^ ^]^ We screened in‐house alpaca nanobody libraries to identify an anti‐MSLN nanobody for the antigen‐binding domain of the CAR construct (KD = 2.88 × 10^−9^ m, Figure S1A, Supporting Information), and an anti‐PD‐1 nanobody for the self‐secreting armory (KD = 5.31 × 10^−10^ m, Figure S1B, Supporting Information). The CAR nanobody recognizes an optimized membrane‐proximal epitope of the MSLN, which we previously found to enhance the anti‐tumor response of CAR‐T cells,^[^ ^20^ ^]^ and has shown strong binding affinity (Figure S1C, Supporting Information, EC50 = 7.06 nm). The anti‐PD‐1 nanobody was functionally comparable to pembrolizumab and nivolumab in competitive PD‐1 binding and blocking downstream signaling, as determined using a dual‐cell luciferase reporter assay (Figure S1D, Supporting Information, EC50 = 2.18 nm). MSLN and PD‐1 nanobodies exhibited high specificity for their target proteins (Figure S1E,F, Supporting Information), with minimal cross‐reactivity with non‐tumor human tissues (Figure S1G, Supporting Information). Tonic signal of the anti‐MSLN VHH CAR was comparable to the commercialized anti‐CD19 scFv CAR, regardless of the gene modification strategy (Figure S1H, Supporting Information).

**Figure 1:**
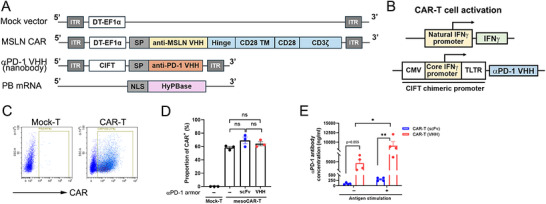
Generation and characterization of NAC‐T cells. A) Schematic diagram showing the structure of the mock vector, PB mRNA, MSLN CAR, and anti‐PD‐1 (αPD‐1) constructs. ITR: *piggyBac* inverted terminal repeats; SP: signal peptide; TM: transmembrane domain; DT‐EF1α/CIFT: promoters for gene expression; NLS: nuclear localization signal; HyPBase: hyperactive *piggyBac* transposase. B) Design of activation‐induced anti‐PD‐1 nanobody production. CIFT: chimeric IFNγ promoter; CMV: cytomegalovirus enhancer; TLTR: Truncated human T‐lymphotropic virus long terminal repeat. C) Representative image of CAR expression evaluated using flow cytometry. D) CAR expression in non‐armored MSLN CAR‐T and MSLN CAR‐T armored with human anti‐PD‐1 scFv‐Fc antibody (scFv) or alpaca‐derived nanobody (VHH). E) αPD‐1 antibody production by MSLN CAR‐T armored with scFv‐Fc antibody or VHH nanobody targeting PD‐1. Shown are the antibody concentrations in the supernatant of the cell culture. Changes with or without MSLN antigen stimulation are shown. Data were collected from three independent donors. Bars indicate mean ± SEM. ^**^ *p* < 0.01; ns, not significant using one‐way ANOVA with multiple comparisons (D) or Welch's *t*‐test (E).

To generate NAC‐T cells, T cells isolated from peripheral blood were co‐electroporated with mRNA encoding hyperactive *piggyBac* transposase, along with plasmids containing anti‐MSLN CAR and anti‐PD‐1 antibody constructs (**Figure** **1**A). The expression of the anti‐PD‐1 nanobody was induced via T‐cell activation, driven by a chimeric promoter that incorporates the core IFN‐γ promoter (Figure 1B).^[^ ^17^ ^]^ Armoring CAR‐T cells with an anti‐PD‐1 antibody did not affect the expression of MSLN CAR, regardless of whether the antibody was an alpaca‐derived nanobody or an scFv‐Fc antibody (the scFv fragment of pembrolizumab with an Fc fragment from human IgG4, Figure 1C,D). NAC‐T cells produced ≈30‐fold higher levels of anti‐PD‐1 agent—both at baseline and after antigen stimulation—compared to CAR‐T cells armored with the scFv‐Fc antibody format (Figure 1E).

We subsequently examined T‐cell subtypes within the NAC‐T cell populations (Figure S2A, Supporting Information). Over 80% of the NAC‐T cells were identified as CD8^+^ cytolytic T cells, similar to those in the MSLN CAR‐T group (Figure S2B, Supporting Information). Given that the proportion of central memory T cells (Tcm) is associated with in vivo CAR‐T cell persistence,^[^ ^21^, ^22^ ^]^ we also analyzed the Tcm subtype and found that the proportion was comparable between NAC‐T and MSLN CAR‐T cells (Figure S2C, Supporting Information). These results suggest that using a nanobody as the armory significantly enhanced the productivity of self‐secreted anti‐PD‐1 antibody, while it did not significantly alter the fundamental characteristics and the stemness of CAR‐T cells.

### 2.2 PD‐1 Blockade by Nanobody Enhanced CAR‐T Expansion and Cytolytic Function

**Figure 2:**
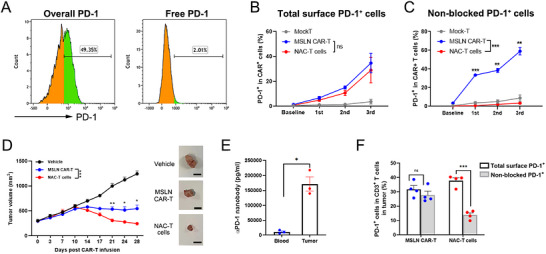
Enhanced in vitro proliferation and anti‐tumor activity of NAC‐T cells. A–C) Self‐secreted nanobodies efficiently block surface PD‐1 on NAC‐T cells. (A) Representative images showing flow cytometry detection of overall PD‐1 (left) and non‐blocked free PD‐1 (right) in NAC‐T cells stimulated with NCI‐H226 tumor cells. In CD3^+^CAR^+^ cells, shown are proportions of B) all cells expressing surface PD‐1 regardless of nanobody blockade and C) cells with non‐blocked PD‐1 available to bind PD‐1 ligands. Flow cytometry analyses were performed after each round of serial tumor stimulation. Mock‐T, MSLN CAR‐T, and NAC‐T from three independent donors were analyzed. D–F) Efficacy of NAC‐T and MSLN CAR‐T was compared in a tumor xenograft mouse model established from the NCI‐H226 human mesothelioma cell line. (D) Tumor growth curve showing significantly reduced tumor volume in NAC‐T cell treated mice. Each group contains six mice. Representative tumor images are shown on the right side. (E) Concentrations of anti‐PD‐1 nanobody in peripheral blood (blue) and tumor tissue lysate (red) were evaluated. (F) Efficient PD‐1 blocking on the surface of intratumoral CD3+ T cells. Single cell suspension of mouse tumor tissue was prepared, and intratumoral CD3^+^ T cells were analyzed using flow cytometry. The percentage of all CD3^+^ cells that express PD‐1, regardless of nanobody blockade (Total surface PD‐1^+^, white bar) was compared with the percentage of cells with non‐blocked PD‐1 (Non‐blocked PD‐1^+^, gray bar). Data are shown as mean ± SEM and analyzed using two‐way ANOVA with multiple comparisons (B–D, F) or unpaired Welch's t‐test (E). ^*^ *p* < 0.05; ^**^ *p* < 0.01; ^***^ *p* < 0.001; ns, not significant.

We next evaluated whether self‐secreted anti‐PD‐1 nanobody could protect CAR‐T cells from functional exhaustion under conditions of persistent antigen exposure. Using a serial tumor stimulation assay with NCI‐H226 cells that highly express MSLN, we assessed total PD‐1 surface level (regardless of nanobody binding) and free PD‐1 surface level that remains unblocked by the nanobody and still available for interaction with immunosuppressive ligands (e.g, PD‐L1). As anticipated, surface PD‐1 of NAC‐T cells was effectively blocked by the self‐secreted nanobody, with minimal unblocked PD‐1 detected (**Figure** **2**A). Under repetitive tumor stimulation, total PD‐1 surface level comparably increased in both MSLN CAR‐T and NAC‐T cells (Figure 2B). However, the proportion of NAC‐T cells bearing non‐blocked PD‐1 remained below 5% during multiple rounds of co‐culture stimulation (3.36 ± 0.76% at the third round, Figure 2C). These results indicate durable, effective PD‐1 blockade in NAC‐T cells, which may contribute to reduced exhaustion and maintained functionality under persistent tumor pressure.

Consistent with durable PD‐1 blockade, we observed a significant increase in the expansion of NAC‐T cells in culture. After five rounds of repetitive tumor cell stimulation, the expansion of MSLN CAR‐T cells was limited during the sixth round of co‐culture, whereas NAC‐T cells continued to proliferate actively. NAC‐T cells yielded up to 24‐fold greater CAR^+^ cell numbers than MSLN CAR‐T cells derived from the same donor (Figure S2D, Supporting Information). Beside the blockade of PD‐1, there were limited changes in the expression of other exhaustion‐related markers such as LAG3, TIM‐3, TCF‐7, and CCR7 (Figure S2E, Supporting Information). This observation is consistent with other preclinical reports,^[^ ^16^, ^23^ ^]^ and may be related to the epigenetic imprint.^[^ ^24^ ^]^ These findings suggest that the self‐secreted anti‐PD‐1 nanobody facilitates the proliferation of MSLN CAR‐T cells under persistent stimulation by tumor cells.

The anti‐tumor activities of MSLN CAR‐T and NAC‐T cells were evaluated using the RTCA assay by co‐culturing them with NCI‐H226 tumor cells at different effector‐to‐target (E:T) ratios. NAC‐T cells showed superior anti‐tumor activity compared with that of CAR‐T cells (Figure S2F, Supporting Information), exhibiting an ≈1.5‐fold increase in cytolytic efficacy at 1:4 and 1:8 E:T ratios (Figure S2G, Supporting Information). These findings indicate the enhanced anti‐tumor activity of NAC‐T cells in vitro.

### 2.3 Targeted Delivery of Anti‐PD‐1 Nanobody Improved Anti‐Tumor Activity of NAC‐T against Solid Tumors

We further investigated the efficacy of NAC‐T in inhibiting tumor growth using rigorous in vivo assays. We enrolled mouse xenograft tumors with much larger volumes (200–300 mm^3^) than those conventionally used in ICI or CAR‐T preclinical studies (30–100 mm^3^).^[^ ^25^, ^26^, ^27^ ^]^ Upon stimulation with NCI‐H226 cells as a model of human malignant mesothelioma (Figure S3A, Supporting Information), we confirmed that NAC‐T cells produced substantial amounts of proinflammatory cytokines, including IFN‐γ, TNF‐α, and IL‐2 (Figure S3B, Supporting Information). We then treated mice bearing NCI‐H226 tumors with a relatively large volume (200 mm^3^) using NAC‐T cells via intravenous administration. NAC‐T treatment achieved complete tumor remission, with excellent tolerability across all dose levels (Figure S3C–F, Supporting Information).

To explore the contribution of the self‐secreted anti‐PD‐1 nanobody, the efficacy of NAC‐T cells was compared with that of conventional MSLN CAR‐T cells in mice bearing even larger NCI‐H226 tumors (300 mm^3^). NAC‐T cells effectively inhibited tumor growth and prevented further progression. MSLN CAR‐T cells showed considerable tumor inhibitory effects; however, the tumors progressed in the long term (Figure 2D; Figure S3C, Supporting Information). The tumor volume in the NAC‐T group was found to be less than half that of the MSLN CAR‐T group at day 28 post‐infusion (242.67 ± 26.66 vs 547.17 ± 69.10 mm^3^), indicating a superior anti‐tumor response from NAC‐T cells.

To assess the in vivo expansion and distribution of NAC‐T cells, we evaluated the number of infused cells in the peripheral blood of tumor‐bearing mice. The T‐cell count and CAR copy number demonstrated enhanced expansion of NAC‐T cells (Figure S3G,H, Supporting Information). Compared to that of MSLN CAR‐T cells, the peak number of total T cells increased approximately three‐fold, and the CAR copy number increased 1.6‐fold in NAC‐T cells. This finding suggests that the self‐secreted anti‐PD‐1 nanobody may influence T‐cell proliferation in both autocrine and paracrine manners.

Regarding tissue distribution, the self‐secreted nanobody was predominantly localized within the tumor rather than circulating in the peripheral blood. The concentration of the anti‐PD‐1 nanobody reached 171 156.82 ± 23 852.03 pg mL^−1^ (Figure 2E), over 6500‐fold higher than that in previous ICI‐armored CAR‐T studies, reporting only 7–26 pg mL^−1^ of self‐secreted non‐nanobody anti‐PD‐1 antibodies detected in tumor tissue.^[^ ^12^, ^16^ ^]^

Analyses of exhausted tumor‐infiltrating T cells revealed a comparable proportion of PD‐1^+^ cells of ≈30% in both NAC‐T and MSLN CAR‐T treated mice (Figure 2F). However, following NAC‐T treatment, the proportion of T cells with non‐blocked PD‐1 drastically decreased to 13.8 ± 1.5%, compared to 27.7 ± 2.7% post MSLN CAR‐T treatment. In the NAC‐T group, receptor occupancy on tumor‐infiltrating T cells reached 63.7 ± 2.6% at day 21, comparable to that with pembrolizumab at 5 mg kg^−1^ dose (mean receptor occupancy ranging from 41.8% at day 1 to 65.4% at day 4, according to the Biologics Licensing Application file of pembrolizumab). These data suggest that self‐secreted nanobodies efficiently block surface PD‐1, promote the expansion of T cells in the TME, and enhance the anti‐tumor activity of NAC‐T cells.

To assess the risk of tumorigenicity in NAC‐T cells, we used ligation target amplification PCR to analyze the genomic integration profile of the *piggyBac* transposon. Ten independently prepared NAC‐T samples were analyzed, and the integration ratio was determined by calculating the number of unique integration sites relative to the total sequenced counts. All samples exhibited an integration ratio of less than 5% without any duplication of the top ten genes, indicating limited integration‐induced tumorigenic risk of NAC‐T cells. The clonal diversity of each sample was evaluated based on the evenness and richness of clones. As shown in the polyclonal–monoclonal distance plane, all samples were mapped to the polyclonal region (Figure S4A, Supporting Information), indicating that the NAC‐T cells were polyclonal. Further analysis revealed evenly distributed integration sites among chromosomes (Figure S4B, Supporting Information) and a lentiviral‐like pattern^[^ ^28^ ^]^ of integration across different gene regions (Figure S4C, Supporting Information). Analyses of common insertion sites did not reveal integration hotspot regions related to tumor or cell proliferation (Table S1, Supporting Information). A soft agar colony formation assay further confirmed that NAC‐T cells were not tumorigenic (Figure S4D, Supporting Information). Overall, the risk of T‐cell tumorigenesis caused by transposon‐related integration was comparable to that of a lentiviral‐based gene modification system.

### 2.4 Design of First‐in‐Human Clinical Trial of NAC‐T Cells and Baseline Patient Characteristics

Building on these promising preclinical results, we sought to explore the safety and efficacy of NAC‐T in patients with malignant mesothelioma in clinical trials. Peripheral blood mononuclear cells (PBMCs) were collected via apheresis to produce NAC‐T cells. T cells were isolated and electroporated with a mixture of transgene‐expressing vectors and *piggyBac* transposon mRNA (Figure 1A). Transduced T cells were cultured in vitro with IL‐7 and IL‐15 for 5–9 days before cryopreservation (see Experimental Section). The NAC‐T cell products from patients showed that the median proportion of CAR^+^ cells in CD3^+^ T cells was 41.9% (range, 11.0–77.9%). The median copy numbers of CAR and anti‐PD‐1 nanobody constructs were 6.1 and 7.0 copies per cell, respectively. The median percentage of the CD4^+^ subpopulation of NAC‐T was 21.1% (range, 8.9–55.8%), and that of the CD8^+^ subpopulation of NAC‐T was 77.3% (range, 26.6–91.1%, Table S2, Supporting Information).

**Figure 3:**
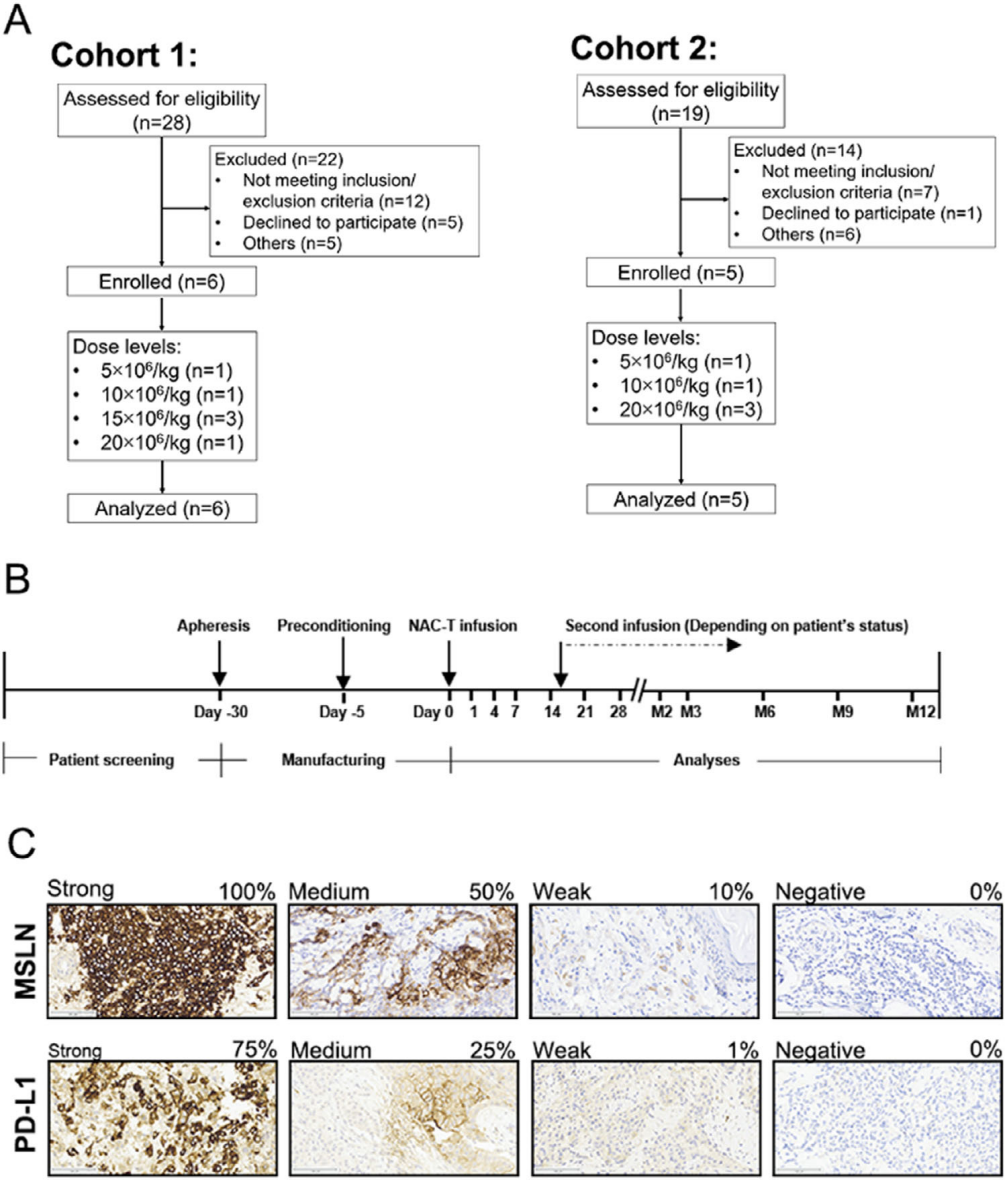
Strategies for patient screening, enrollment, and treatment. A) CONSORT flow diagram. B) Treatment scheme of clinical study. C) Inclusion criteria were determined using IHC staining against MSLN and PD‐L1. Shown are representative images of a patient tumor with strong, medium, weak, and negative staining. Numbers indicate the percentage of tumor cells expressing MSLN or PD‐L1. Patients were enrolled when more than 50% of tumor cells expressed MSLN expression (e.g, strong or medium) and more than 1% cells express PD‐L1 (e.g, strong, medium or weak) were detected in FFPE tumor sections. Scale bar, 100 µm.

To explore the safety and efficacy of NAC‐T therapy, we first conducted a preliminary exploratory dose‐escalating investigator‐initiated trial at two centers (cohort 1). From September 1st 2020 to November 30th 2022, 28 patients with malignant mesothelioma were assessed for eligibility, and six patients were finally enrolled (BZ‐01–BZ‐06). Four dose levels (DLs) were designed: 5 × 10^6^ per kg (DL1, n = 1), 10 × 10^6^ per kg (DL2, n = 1), 15 × 10^6^ per kg (DL3, n = 3), and 20 × 10^6^ per kg (DL4, n = 1). With encouraging results regarding the efficacy and safety of the exploratory phase, we synchronously initiated another dose‐escalating cohort (cohort 2). From January 1st 2022 to September 31st 2022, 19 patients with malignant mesothelioma were assessed and five patients were finally enrolled (BZ‐07–BZ‐11). Three DLs were designed: 5 × 10^6^ per kg (DL1, n = 1), 10 × 10^6^ per kg (DL2, n = 1), and 20 × 10^6^ per kg (DL4, n = 3). The CONSORT diagrams of these two cohorts are shown in **Figure** **3**A. Separate and combined results for these two cohorts are reported in the following sections.

All patients underwent apheresis 30 days before infusion, received lymphodepletion with a regimen based on 30 mg m^−2^ cyclophosphamide and 300 mg m^−2^ fludarabine for 3 days, and then received an intravenous infusion with a pre‐designed dose of NAC‐T cells. The primary endpoints were tolerability and dose‐limiting toxicity of NAC‐T treatment. The secondary endpoint was efficacy observations of the ORR, with exploratory endpoints including progression‐free survival (PFS) and OS. For pharmacokinetic and pharmacodynamic analyses, peripheral blood samples were collected on days 1, 4, 7, 14, 21, 28, and later specified time points throughout the follow‐up process (Figure 3B). The copy number of the CAR transgene, concentration of anti‐PD‐1 nanobody, and levels of inflammatory cytokines in the peripheral blood were assessed. Repeated infusion of NAC‐T cells was allowed, depending on the decision of clinical investigators. Four patients received a second infusion on days 14 (BZ‐03), 35 (BZ‐01 and BZ‐05), and 42 (BZ‐02) with the same or escalated dose of NAC‐T (Table S3, Supporting Information). Overall, 11 patients from the two cohorts were eligible for treatment response evaluation.

The baseline characteristics of the patients are listed in **Table** **1** and Table S3 (Supporting Information). The inclusion criteria for antigen expression in tumor cells were over 50% for MSLN and positivity for PD‐L1 (Figure 3C). For all 11 patients, medium to strong levels of MSLN expression were observed in over 90% of tumor cells. PD‐L1 expression was observed in at least 10% of tumor cells in 66.7% (4 of 6) of the patients in cohort 1 and 80% (4 of 5) of the patients in cohort 2. Nine out of 11 (81.8%) patients presented disease at an advanced stage.^[^ ^29^ ^]^ These patients were heavily treated, with a median of three prior regimens (range, 1–9).

**Table 1:** Baseline characteristics of all 11 patients.

Characteristics	Cohort 1 N=6, n [%]	Cohort 2 N=5, n [%]	Overall N=11, n [%]
Age, median (min, max), year	64 (53, 66)	48 (32, 59)	59 (32, 66)
Sex			
Male	0	4 (80)	4 (36)
Female	6 (100)	1 (20)	7 (64)
ECOG status			
0	2 (33)	0	2 (18)
1	4 (67)	5 (100)	9 (82)
Clinical stage, MPM			
I‐II	0	0	0
III	1 (17)	1 (20)	2 (18)
IV	3 (50)	0	3 (27)
Clinical stage, MPeM			
I	0	0	0
II	0	2 (40)	2 (18)
III	2 (33)	2 (40)	4 (36)
Total tumor measurement, median (min, max), mm	43.0 (26.0, 73.5)	131.3 (100.3, 260.5)	73.5 (26.0, 260.5)
MSLN expression			
90‐100%	6 (100)	5 (100)	11 (100)
PD‐L1 expression			
>50%	3 (50)	1 (20)	4 (36)
10‐50%	1 (17)	3 (60)	4 (36)
<10%	2 (33)	1 (20%)	3 (27)
Prior surgery	3 (50)	4 (80)	7 (64)
Prior regimens, median (min, max)	2.5 (1, 7)	4 (2, 9)	3 (1, 9)
Chemotherapy	6 (100)	5 (100)	11 (100)
Anti PD‐1/PD‐L1 therapy	1 (17)	1 (20)	2 (18)
Anti‐angiogenesis	4 (67)	3 (60)	7 (64)

Abbreviations: ECOG, Eastern Cooperative Oncology Group performance score;

PD‐L1, programmed death ligand 1.

### 2.5 NAC‐T Treatment was Safe and Effective

All 11 treated patients (100%) experienced treatment‐related adverse events (TRAEs), with the most common being cytokine release syndrome (CRS), leukopenia, increased IL‐6, and fever, each occurring in 73% of the patients (**Table** **2**). The most common TRAE that over grade 3 were leukopenia (64%), followed by neutropenia (45%), lymphopenia (45%), anemia (18%), and thrombocytopenia (18%) (Table S4, Supporting Information), which were most likely related to lymphodepletion chemotherapy as expected. CRS was observed in 8 of 11 (73%) patients, with 5 of 6 (83%) in cohort 1 and 3 of 5 (60%) patients in cohort 2. All CRS‐related events were below grade 3, and the patients recovered within five days. No dose‐limiting toxicity was observed at any dose level. Overall, NAC‐T treatment was well tolerated in patients with malignant mesothelioma.

**Table 2:** Treatment related adverse events (TRAEs) were observed in over 10% patients.

TRAEs, N [%]	Cohort 1 (N=6)		Cohort 2 (N=5)		Overall(N=11)	
	Any grade	≥ Grade 3	Any grade	≥ Grade 3	Any grade	≥ Grade 3
**Immune system disorders**						
CRS	5 (83)	0	3 (60)	0	8 (73)	0
**Blood any lymphatic system disorders**						
Leukopenia	4 (67)	4 (67)	4 (80)	3 (60)	8 (73)	7 (64)
Neutropenia	5 (83)	4 (67)	2 (40)	1 (20)	7 (64)	5 (45)
Anemia	6 (100)	2 (33)	1 (20)	0	7 (64)	2 (18)
Lymphopenia	4 (67)	4 (67)	2 (40)	1 (20)	6 (55)	5 (45)
Thrombocytopenia	4 (67)	1 (17)	2 (40)	1 (20)	6 (55)	2 (18)
**Investigations**						
Increased IL‐6	4 (67)	0	4 (80)	1 (20)	8 (73)	1 (9)
Increased CRP	4 (67)	0	3 (60)	0	7 (64)	0
Increased ALT	1 (17)	0	2 (40)	0	3 (27)	0
Increased AST	1 (17)	0	1 (20)	0	2 (18)	0
**General disorders**						
Fever	5 (83)	1 (17)	3 (60)	0	8 (73)	1 (9)
Malaise	5 (83)	0	0	0	5 (45)	0
Chills	3 (50)	0	0	0	3 (27)	0
Pain	2 (33)	0	0	0	2 (18)	0
**Metabolism and nutrition disorders**						
Hypoalbuminemia	5 (83)	0	1 (20)	0	6 (55)	0
Anorexia	3 (50)	0	0	0	3 (27)	0
**Vascular disorders**						
Hypotension	5 (83)	0	0	0	5 (45)	0
**Cardiac disorders**						
Sinus tachycardia	2 (33)	0	2 (40)	0	4 (36)	0
**Skin and subcutaneous tissue disorders**						
Rash	3 (50)	0	0	0	3 (27)	0
**Respiratory disorders**						
Pneumonitis	2 (33)	0	1 (20)	1 (20)	3 (27)	1 (9)
Dyspnea	3 (50)	0	0	0	3 (27)	0
**Gastrointestinal disorders**						
Nausea	2 (33)	0	1 (20)	0	3 (27)	0
Vomiting	2 (33)	0	0	0	2 (18)	0
Abdominal distension	1 (17)	0	1 (20)	0	2 (18)	0
**Infections and infestations**						
Conjunctivitis	0	0	2 (40)	0	2 (18)	0

*Note*: TRAEs include events that were definitely, probably, and possibly related to CAR‐T therapy.

Adverse events were graded according to CTCAE v5.0, CRS was graded according to ASTCT criteria. CRS, cytokine release syndrome.

**Figure 4:**
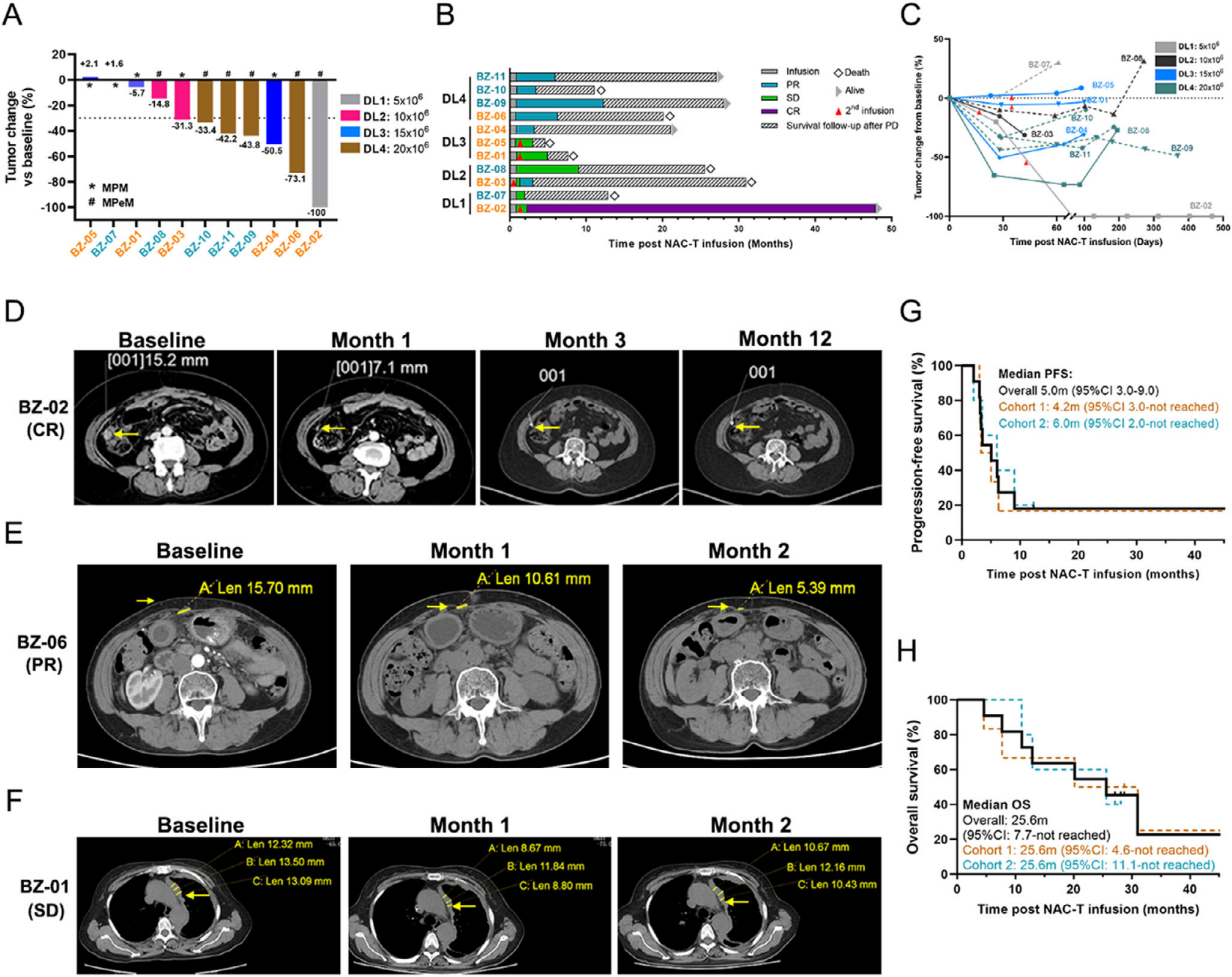
Outcome of NAC‐T treatments in patients with mesothelioma. A–C) Anti‐tumor activity of NAC‐T shown by (A) changes of total tumor measurement as the best response, (B) overall response and survival time of each patient, and (C) tumor change over time at indicated time points post treatment. Data from both cohort 1 (yellow) and cohort 2 (blue) are shown. Different colors indicate patients enrolled in each dose level or in different cohorts. Each bar or line represents an individual patient. Length of colored or shadowed bars in (B) indicates duration of each response or survival status. Red triangles indicate the time of the second infusion. D–F) Representative CT images show tumor reduction in (D) the patient with CR, BZ‐02, (E) a patient with PR, BZ‐06, and (F) a patient with SD, BZ‐01. Arrows indicate tumor lesions, and numbers indicate tumor measurements. CR: complete response; PR: partial response; SD: stable disease. G,H) Kaplan–Meier analyses on (G) progression‐free survival (PFS) and (H) overall survival (OS). Solid lines represent overall survival data from all 11 patients. Dotted lines indicate survival data from cohort 1 (yellow) or cohort 2 (blue).

Treatment response was evaluated according to RECIST v1.1, and mRECIST was applicable for MPM. For all 11 patients, the overall ORR was 63.6% (7/11). One patient achieved a complete response (CR), six patients achieved a partial response (PR), and four patients achieved stable disease (SD) as the best responses. The DCR was 100% (11/11). The separate response rates of the two cohorts were 66.7% (4/6) and 60.0% (3/5) (**Figure** **4**A–C). A higher ORR was observed in patients with MPeM than in those with MPM (83.3% vs 40.0%), although not statistically significant due to the small sample size (6 vs 5 cases, *p* = 0.24 based on Fisher's exact test). Neither prior treatment with PD‐1/PD‐L1 inhibitors nor PD‐L1 expression levels correlated with efficacy (Table S3, Supporting Information).

A significant reduction in the tumor load was observed in most patients after NAC‐T treatment (Figure 4A). Patient BZ‐02 achieved a durable CR, which lasted 45.6 months (Figure 4B). The tumor decreased from 15.2 to 7.1 mm in the first month and disappeared by month 3 (Figure 4D). Most recently, the CR status was confirmed on October 12th 2024, 47.9 months after the first infusion. The patient is alive at the time of manuscript preparation. As a representative case of patients with PR, BZ‐06 achieved notable tumor reduction from 15.7 mm at baseline to 10.6 mm after one month, with the size further decreasing to 5.4 mm at month 2 (Figure 4E). As a representative case of patients with SD, BZ‐01 showed only a modest decrease in tumor thickness from baseline to month 2 (Figure 4F).

By the data cut‐off date on November 29th, 2024, the median follow‐up time for cohorts 1 and 2 was 24.5 (range, 4.6–47.9) and 25.6 months (range, 11.1–28.1), respectively. The median PFS and OS of cohort 1 were 4.2 (95% CI, 3.0 – not reached) and 25.6 months (95% CI, 4.6 – not reached), respectively, and those of cohort 2 were 6.0 (95% CI, 2.0 – not reached) and 25.6 months (95% CI, 11.1 – not reached), respectively (Figure 4G,H). For all 11 patients, the overall median PFS was 5.0 months (95% CI, 3.0 – 9.0) and the median OS was 25.6 months (95% CI, 7.7 – not reached). Taken together, these findings demonstrate the substantial anti‐tumor activity of NAC‐T in patients with malignant mesothelioma.

### 2.6 Expansion and Persistence of NAC‐T in Patients

**Figure 5:**
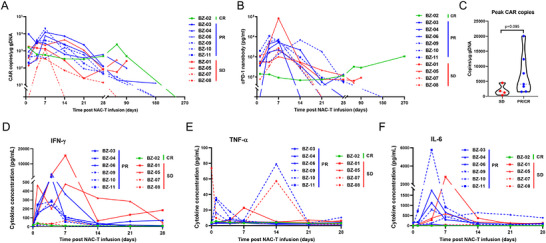
Dynamics of NAC‐T cells and related cytokines in the peripheral blood of 11 patients. A,B) Dynamics of NAC‐T cells are represented by (A) MSLN CAR gene copy number and (B) concentration of anti‐PD‐1 nanobody at the indicated time post infusion. C) Peak value of CAR copies was compared between patients with PR/CR versus patients with SD. Welch's *t*‐test was used for statistical analysis. D–F) Changes in cytokines related to NAC‐T function, including (D) IFN‐γ, (E) TNF‐α, and (F) IL‐6. Each line represents one patient, and data are categorized as red (SD), blue (PR), or green (CR) according to the best responses. Data are shown in cohort 1 (solid line) and cohort 2 (dotted line).

We evaluated the expansion and persistence of NAC‐T cells in these patients, which intuitively represented the anti‐tumor effects of such therapies on solid tumors. In the peripheral blood, the CAR copy number and concentration of the anti‐PD‐1 nanobody increased, reaching a peak value (Cmax) within 14 days post‐infusion (**Figure** **5**A,B). A modest increase in the peak level of CAR gene copies was observed in patients with PR/CR compared to that of patients with SD, suggesting a potential correlation between NAC‐T cell expansion and anti‐tumor activity, although the difference was not statistically significant (*p* = 0.095, Figure 5C). Prolonged NAC‐T cell persistence was observed in the patient with CR (BZ‐02). After the first infusion, the CAR copy number was only modestly increased, reaching a peak level of 1596 copies per µg gDNA. Interestingly, after the second infusion, re‐infused NAC‐T cells continued to expand and reached another peak at day 60 (2353 copies per µg, Figure 5A). A similar re‐peaking of the CAR copy number was observed in another patient, BZ‐01, who received a second infusion on day 35. A substantial level of anti‐PD‐1 nanobodies (290–1066 pg mL^−1^) was observed in the peripheral blood of BZ‐02 but not BZ‐01, even three months after the initial infusion (Figure 5B). Since a large proportion of NAC‐T cells are localized in the tumor microenvironment rather than in peripheral blood, as observed in mouse models (Figure S3I, Supporting Information), the sustained production of the anti‐PD‐1 nanobody suggests long‐lasting NAC‐T cell persistence, possibly at the tumor site, contributing to a durable CR that has lasted 45.6 months.

To investigate the timing and intensity of NAC‐T cell activation, the dynamics of key inflammatory cytokines were monitored after NAC‐T cell infusion. In most patients, significant increases in IFN‐γ, TNF‐α, and IL‐6 levels were observed during the first two weeks post‐infusion (Figure 5D–F), suggesting activation‐induced cytokine production of NAC‐T cells. Consistent with observations in other clinical trials of CAR‐T therapy, patients with CRS showed significantly increased peak levels of IL‐6 (*p* = 0.0242 with Mann–Whitney U test), demonstrating the importance of IL‐6 in CRS management.

The immunogenicity of MSLN CAR and anti‐PD‐1 nanobodies was also evaluated by measuring the levels of anti‐drug antibodies (ADAs) in the peripheral blood of patients. No pre‐existing ADAs against the MSLN CAR construct were observed, whereas pre‐existing ADAs against the anti‐PD‐1 nanobody were observed in 36.4% of patients (4 out of 11, Table S5, Supporting Information). Considering treatment‐emergent ADAs, only one patient (10%) showed a high level (titer ≥ 1000) of ADAs against CAR and anti‐PD‐1 nanobody, respectively. In further blocking assays, patient serum did not impair the binding of PD‐1 to PD‐L1, regardless of ADA positivity or ADA titer (Figure S5, Supporting Information). These results demonstrated that the presence of ADA had a limited impact on NAC‐T cell expansion.

### 2.7 Expansion of TAA‐Responsive Bystander T Cells in Patients that Achieved PR

**Figure 6:**
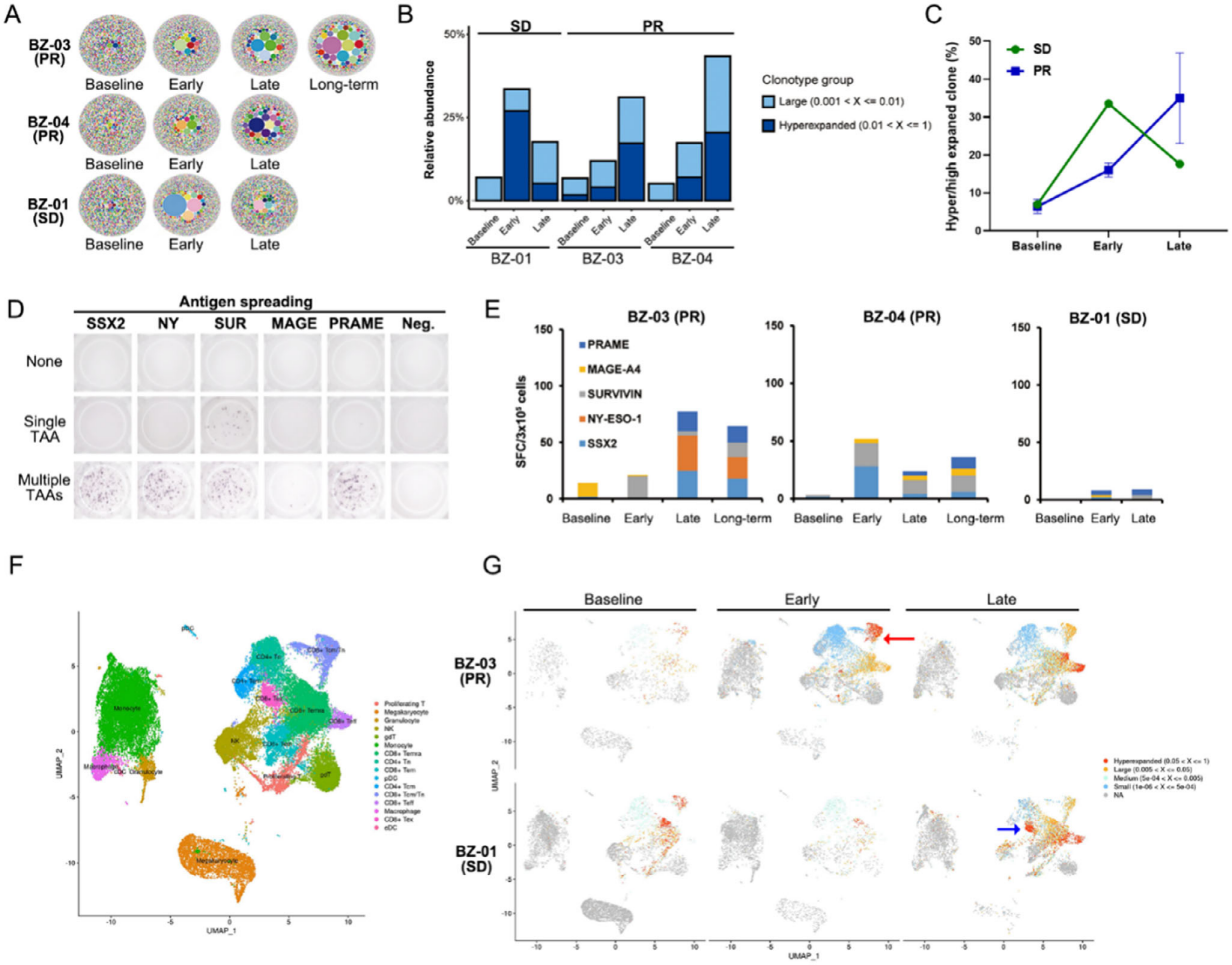
NAC‐T infusion triggers systemic endogenous anti‐tumor T‐cell response in patients. A) Top 10 000 TCR clones are shown using packed circles in each sample from three individual mesothelioma patients (each circle represents a T‐cell clone, and circle size represents clonal fraction). B) Relative abundance of TRB across three patients clustered by their clinical outcomes. Each patient has three bars representing samples collected at different time points after infusion. C) Change in large and hyperexpanded T‐cell groups after infusion. D,E) Antigen spreading assay identified a sustained increase of anti‐tumor T cells against non‐MSLN TAAs in the peripheral blood of a patient with PR but not a patient with SD. (D) Representative images of patient PBMC sample showing IFN‐γ producing cells in response to indicated TAAs. (E) Cumulative number of IFN‐γ producing cells (spot‐forming cells, SFC) in patient PBMCs at different time points post treatment was measured using IFN‐γ ELISpot upon stimulation of each indicated TAA. SSX2: synovial sarcoma X2; NY: NY‐ESO‐1; SUR: Survivin; PRAME: preferentially expressed antigen of melanoma; MAGE: MAGE‐A4, melanoma‐associated antigens family A4. F) UMAP plot showing 43 974 cells from six samples, which were divided into 16 main clusters. Each dot represents one single cell, and color corresponds to different cell populations. G) UMAP plot showing the clonal expansion level of patients BZ‐03 and BZ‐01. Each dot corresponds to a single cell, and colors represent different levels of clonal expansion at different times. For all patient samples, early represents days 11–14, late represents day 28, and long term represents days 42–60, depending on sample accessibility. Colored arrows indicate hyperproliferating cells.

To understand the mechanism underlying NAC‐T function, we conducted TCR beta chain (TRB) sequencing in three patients (two with PR and one with SD). The results showed a continuous increase in the total frequency of hyperexpanded TRB clonotypes in the PR cases (BZ‐03 and BZ‐04), whereas the SD case (BZ‐01) exhibited only transient expansion of hyperexpanded TRB clones (**Figure** **6**A–C). A Sankey diagram illustrates the frequency and proportion of the top 15 expanded TRB clones (Figure S6A, Supporting Information). Repertoire overlap analyses indicated the presence of shared TRB clones among samples from the same patient and across different patients, suggesting similarities in the antigen recognition pattern of the expanded clones (Figure S6B, Supporting Information). By detecting IFN‐γ production upon stimulation of tumor‐associated antigens (TAAs), we further analyzed T cell responsiveness against five commonly expressed TAAs: synovial sarcoma X2, NY‐ESO‐1, Survivin, preferentially expressed antigen of melanoma, and melanoma‐associated antigens family A4.^[^ ^30^, ^31^, ^32^ ^]^ The expression of these TAAs in mesothelioma was validated by comparing tumor biopsies with PBMCs from the same patient (BZ‐03, Figure S6C, Supporting Information). When stimulated with a peptide pool of the indicated TAAs, IFN‐γ producing T cells were detected in patient peripheral blood after infusion, demonstrating the responsiveness of bystander T cells against one or multiple TAAs (Figure 6D,E). Consistent with the T cell clonal expansion observed in TCR sequencing, patients with PR showed higher and continuous expansion of TAA‐responsive T cells than those of patients with SD. While endogenous T cell expansion has been hypothesized in a previous study,^[^ ^6^ ^]^ our findings provide the first experimental evidence that such T cells can be activated by specific tumor antigens.

We also performed single‐cell sequencing using PBMC samples from a patient with PR (BZ‐03) and a patient with SD (BZ‐01). Sequencing data revealed the presence of 16 immune cell subpopulations, particularly T cells with distinct memory phenotypes distinguished by the expression of surface markers (Figure 6F). We further plotted the frequency of TCR clonotypes to investigate the phenotype of the hyperexpanded T cells and their dynamics over time (Figure 6G). As anticipated, the most expanded T‐cell clones were found in the CD8^+^ Temra and CD8^+^ Teff populations, as a shared feature of both patients with PR and SD. Interestingly, a highly expanded CD8^+^ Tcm/Tn population was observed in the patient with PR, especially at early post treatment time points (red arrow). In contrast, the most expanded T‐cell clones were in the same CD8^+^ subpopulation but soon became exhausted after infusion (blue arrow) in the patient with SD, consistent with the relatively lower level of anti‐PD‐1 nanobody (Figure 5B). These findings suggest that a substantial amount of anti‐PD‐1 nanobody produced by NAC‐T cells may facilitate the expansion of CAR^+^ cells in an autocrine manner and help prevent the exhaustion of bystander anti‐tumor T cells in a paracrine manner (Figure S7, Supporting Information).

## 3 Discussion

As a living cell therapy, CAR‐T cells can be equipped with cytokines, receptors, antibodies, or antibody‐like proteins to augment their anti‐tumor efficacy.^[^ ^33^ ^]^ In this study, we developed MSLN‐targeting CAR‐T cells and armored them with an alpaca‐derived anti‐PD‐1 nanobody. Armored CAR‐T cells (also known as NAC‐T cells) exhibit enhanced tumor cytotoxicity in cell cultures and mouse xenograft tumor models. Designed to secrete anti‐PD‐1 nanobodies upon activation, NAC‐T cells were capable of delivering over 170 000 pg mL^−1^ PD‐1 blocking nanobodies into solid tumors, enabling the blockade of over 60% surface PD‐1 on tumor‐infiltrated T cells. In the subsequent first‐in‐human phase I dose‐escalation study, we evaluated the safety profile and anti‐tumor activity of NAC‐T therapy in patients with MSLN‐positive malignant mesothelioma. Clinical benefits were observed among all 11 patients treated across four dose levels of NAC‐T cells in this study, including one CR, six PR, and four SD as the best responses. The overall ORR reached 63.6%, the DCR was 100%, the median PFS was 5.0 months (95% CI, 3.0–9.0), and the median OS was 25.6 months (95% CI, 7.7 – not reached), greatly surpassing previous studies focused on second‐line treatment for malignant mesothelioma with the median OS limited to ≈11 months and an ORR below 22%, including nivolumab,^[^ ^34^ ^]^ pembrolizumab,^[^ ^35^ ^]^ and MSLN‐targeting T‐cell therapies.^[^ ^6^, ^7^, ^8^, ^11^, ^36^, ^37^ ^]^ Thus, as a proof‐of‐concept, this study is the first to demonstrate improved clinical activity of nanobody‐armored CAR‐T by ameliorating T‐cell exhaustion, which offers NAC‐T therapy as an emerging treatment option for patients with malignant mesothelioma.

As reported in clinical trials over the last decade, most MSLN‐targeting CAR‐T cell therapies are generally safe yet have limited efficacy in treating solid tumors such as malignant mesothelioma. June et al. explored the clinical activity of mRNA or lentivirus‐transduced MSLN‐targeting CAR‐T cells in different trials,^[^ ^6^, ^7^, ^8^ ^]^ exhibiting only one PR that lasted no more than 6 months in six patients; in other enrolled patients with other tumor types, such as ovarian or pancreatic cancers, the best response was only SD. Sadelain et al. combined intrapleural MSLN CAR‐T cell infusion with subsequent pembrolizumab administration after 6 weeks (range, 4–17 weeks).^[^ ^11^ ^]^ In 16 patients receiving at least three doses of pembrolizumab (range, 1–30 doses) with at least 3 months of follow‐up after the third dose, the best response was two PRs with complete metabolic responses, with an ORR of 12.5% and a median OS of 23.9 months. Most recently, a CAR‐T‐like MSLN‐targeting T‐cell receptor fusion construct (TRuC, Gavo‐cel) T‐cell therapy was evaluated in patients with malignant mesothelioma, with the best response was four PRs out of 19 evaluable patients, yielding an ORR of 21% with a median PFS of 5.6 months and a median OS of 11.2 months.^[^ ^36^ ^]^ In addition, a clinical study on non‐CAR‐T recombinant immunotoxin targeting MSLN has reported an ORR of 30% in 10 patients; however, the longest survival time was below 19 months.^[^ ^38^ ^]^ Compared with these previous studies on non‐armored MSLN CAR‐T cells, our NAC‐T therapy substantially improved the clinical outcomes of patients with malignant mesothelioma.

Engineering CAR‐T cells to secrete antibodies, cytokines, or fusion proteins has been widely studied in preclinical research. Examples include PD‐1 or PD‐L1 blocking antibodies,^[^ ^12^, ^13^, ^15^, ^16^, ^39^, ^40^, ^41^, ^42^, ^43^ ^]^ cytokines such as IL‐7,^[^ ^44^ ^]^ or bispecific trap protein co‐targeting PD‐1 and TGF‐β.^[^ ^45^ ^]^ In preclinical studies, these approaches enhanced T‐cell expansion and effector function, and increased CAR‐T infiltration and persistence, suggesting the potential to induce durable tumor remission. A cytokine‐armored MSLN CAR‐T that self‐secretes IL‐7 and CCL19 (7 × 19 CAR‐T) has been tested in two patients with pancreatic or ovarian cancers. With five infusions, the best response was CR after 8 months.^[^ ^46^ ^]^ To the best of our knowledge, our group is the first reporting the clinical benefits of ICI antibody armored CAR‐T cells. In our previous trial (NCT03615313), ten patients were treated with MSLN CAR‐T cells that self‐secrete anti‐PD‐1 scFv antibody, and the ORR was 20%.^[^ ^47^ ^]^ One patient with ovarian cancer achieved PR and survived for over 17 months.^[^ ^19^ ^]^ Therefore, the improved clinical efficacy of NAC‐T can be attributed to the armored anti‐PD‐1 nanobodies.

The NAC‐T therapy demonstrates promising anti‐tumor activity and favorable tolerability in patients, with the best response being a durable CR that lasted 45.6 months. In patient BZ‐02, CAR expression became undetectable by month 9 despite persistent anti‐PD‐1 nanobody in peripheral blood. Mouse model data (Figure 2E; Figure S3I, Supporting Information) revealed substantial accumulation of NAC‐T cells and secreted anti‐PD‐1 nanobody within the tumor microenvironment, indicating tumor‐localized persistence and continued ICI production even after circulating CAR‐T cells decline. Regarding safety, the patient with the highest IL‐6 peaking level (BZ‐11) experienced increased body temperature of 38.5 °C and grade 1 CRS in the first week post‐infusion. Tocilizumab and antipyretics were administered, and the symptoms had recovered by the next day.

In this study, we have not observed significantly reduced CAR‐T expansion or impaired clinical response of NAC‐T cells in patients with ADA. For instance, ADA positivity was observed in patient BZ‐02, who achieved the deepest and durable response and showed a persistent detectable level of anti‐PD‐1 antibody. Patient BZ‐10 exhibited high ADA levels against the anti‐PD‐1 nanobody, yet achieved the third‐highest peak nanobody concentration. Furthermore, serum from this patient showed little evidence of blocking the interaction between PD‐1 and PD‐L1 (Figure S5, Supporting Information). In consistent, many commercial CAR‐T products have reported ADA positive in clinical trials, and the incidence may vary (according to the Biologics Licensing Application files). For tisa‐cel, the incidence of pre‐ADA was 66–94%, and that of treatment‐induced ADA was 9–33%. Other approved CAR‐T cell therapies reported ADA incidence from 1% (liso‐cel, trial TRANSFORM) to 47% (ide‐cel, trial KarMMa). Overall, our current data suggest that ADA has a limited impact on the clinical response of NAC‐T therapy.

In this study, the secretion of anti‐PD‐1 nanobodies by NAC‐T cells in the TME promoted the expansion of tumor‐infiltrating T cells, potentially inducing effects similar to those reported for PD‐1 blocking therapy. The NAC‐T treatment significantly increased PD‐1 inhibitor levels at the local tumor site, compared with previously reported CAR‐T cells that self‐secrete PD‐1 antibodies in scFv or full‐length IgG formats.^[^ ^12^, ^16^ ^]^ The elevated intratumoral PD‐1 antibody levels may be crucial for the epitope spreading of T cells, which induces persistent clonal expansion of anti‐tumor T cells, enhanced immune responses against tumor cell death,^[^ ^48^ ^]^ and a second wave of endogenous anti‐tumor activity (Figure S7, Supporting Information, graphical abstract). Epitope spreading may be associated with better clinical outcomes after CAR‐T treatment;^[^ ^6^, ^11^ ^]^ however, limited real‐world evidence was reported before this study.

## 4 Limitations

This study has some limitations. First, the sample size was small, owing to its first‐in‐human nature and the inclusion of only patients with malignant mesothelioma with relatively high MSLN expression (exceeding 50%). The limited patient cohort also poses challenges for adequately estimating the immunogenicity of NAC‐T cells. To address this, we will enroll more patients diagnosed with various solid tumors and a wider range of MSLN expression levels in future studies. Second, the secretion of anti‐PD‐1 nanobody relies on T‐cell activation, which may vary among patients and pose challenges for control and standardization. Future investigations will therefore focus on elucidating the factors influencing NAC‐T activation and nanobody production, aiming to identify potential biomarkers for assessing efficacy and safety. Additionally, our current mechanistic study focused on the bulk peripheral T cells due to the low abundance of peripheral NAC‐T cells detected in patients with solid tumor. Further improvements will aim to optimize the NAC‐T expansion in vivo and refine the analyses for pre‐enriched NAC‐T cells. Furthermore, a head‐to‐head comparison of NAC‐T against a combination of CAR‐T with systemic ICI was not included due to the short half‐life of the anti‐PD‐1 nanobody in vivo. Such inherent pharmacological properties of native nanobody present challenges for separate clinical administration unless engineered, thus defining NAC‐T as an integrated, standalone therapy by design. Although this study established NAC‐T as a monotherapy, its design and preliminary profile provide a foundation for future comparisons and explorations of potential combination strategies. These efforts are anticipated to deepen our understanding of the underlying biology of different types of armored CAR‐T cells and promote their clinical translational applications.

## 5 Conclusion

In conclusion, based on robust preclinical research findings, this first‐in‐human study of NAC‐T cell therapy demonstrated favorable profiles of safety, tolerability and efficacy, leading to clinical benefits observed in all 11 patients who received NAC‐T treatment. These findings underscore the potential of this therapeutic strategy for managing MSLN^+^/PD‐L1^+^ malignant solid tumors, shedding light on future large‐scale clinical trials. In addition, we have been continuously making efforts to enhance the expansion, persistence, and anti‐tumor functionality of NAC‐T cells, wishing to provide a more efficient and practical therapeutic approach and ultimately pave the way for transformative advancements in future cancer treatment.

## 6 Experimental Section

### Clinical Trial Design

This study was a phase I, open‐label, single‐arm, multicenter, investigator‐initiated clinical trial designed to evaluate the safety and efficacy of NAC‐T (also named as BZDS1901) in the treatment of solid tumors, such as malignant mesothelioma (MM), in two cohorts. Cohort 1 was approved by the ethics committees of Shanghai Mengchao Cancer Hospital of Shanghai University (NCT05373147) and Shanghai Tenth People's Hospital (NCT04503980). Cohort 2 was approved by the ethics committee of the National Cancer Center (NCT05089266). A written informed consent form was signed by patients before enrollment. Patients may be eligible for receiving an additional dose at least two weeks after the first infusion at the investigator's discretion. Imaging assessments to evaluate treatment responses were carried out on day 28 post the first infusion. The primary study endpoint was the incidence of adverse events (AEs) and dose‐limiting toxicity. The investigators were responsible for monitoring and reporting treatment‐related AEs through 12 months post‐infusion. AEs were graded according to the NCI Common Terminology Criteria for Adverse Events (CTCAE) Version 5.0. Grading of CRS and immune effector cell‐associated neurotoxicity syndrome (ICANs) was based on the American Society for Transplantation and Cellular Therapy (ASTCT) consensus grading system. The other endpoints focused on treatment responses, including ORR, PFS, OS, tumor reduction, dynamics of NAC‐T cells, and critical cytokines.

### Cell Lines

The human mesothelioma cell line NCI‐H226 (RRID: CVCL_1544) was purchased from the American Type Culture Collection (ATCC). NCI‐H226 cells were modified to stably overexpress the human PD‐L1 gene by lentiviral transfection. The modified cell line has been authenticated and confirmed free of mycoplasma contamination (see “Cell Line Authentication Report” and “Cell Mycoplasma Detection Report”). Tumor cell lines were cultured in Dulbecco's modified Eagle medium (DMEM, Gibco, Cat#11965118) containing 10% fetal bovine serum (FBS, Gibco) at 37 °C with 5% CO_2_.

### Generation of Anti‐MSLN CAR and Anti‐PD‐1 Nanobody Expression Vectors

Synthesized peptides of human MSLN and PD‐1 (10 µg mL^−1^) were used to immunize alpacas, followed by an immune boosting one month later. Phage display was used to screen out the nanobody against MSLN for the CAR construct and the nanobody against PD‐1 for the armor.

### Manufacturing CAR‐T and NAC‐T Cells

Fresh PBMCs from healthy donors and patients were collected by apheresis. T cells were enriched through magnetic separation using anti‐CD4 microbeads (Miltenyi Biotec, Cat#170‐076‐702) and anti‐CD8 microbeads (Miltenyi Biotec, Cat#170‐076‐703) and activated with anti‐CD3 antibody (Miltenyi Biotec, Cat#170‐076‐116) and 4‐1BBL antigen (Acro, Cat#41L‐H5256, 5 µg mL^−1^) coated T175 flasks. After 2 days of T cell stimulation, a mixture of *piggyBac* transposase mRNAs, CAR plasmid, and PD‐1 armor plasmid was co‐transduced by electroporation into T cells using the GT Flow Transfection System (MaxCyte) according to the manufacturer's instructions. After electroporation, cells were transferred to AIM‐V culture medium (Thermo Fisher Scientific, Cat#A38308‐01) supplemented with CTS Immune Cell Serum Replacement (Thermo Fisher Scientific, Cat#A25961‐01), IL‐7 (Primer Gene, Cat#GMP‐101‐07), and IL‐15 (Primer Gene, Cat#GMP‐101‐15) for 5–9 days. When T cells reached the number required for clinical dosage, a sample of the cultured T cells was taken for quality control, and the remaining cells were collected and aliquoted to CryoStore freezing bags (Origen Biomedical, Cat#CS50) containing cryopreserved solution (with 10% dimethyl sulfoxide). The bags were placed in a controlled‐rate CryoMed Freezer 7451 (Thermo Fisher Scientific) for cryopreservation.

### CAR‐T Expansion and Cytotoxicity

To evaluate long‐term proliferation under tumor stimulation, mock‐T, MSLN CAR‐T, and MSLN NAC‐T cells derived from the same donor were co‐cultured with the NCI‐H226 cell line. The proliferation of tumor cells in the co‐culture system was inhibited by pre‐treatment with 25 µg mL^−1^ mitomycin C for 2–4 h. On day 0, CAR^+^ cells were counted and seeded in a 6‐well plate at the indicated effector‐to‐target (E:T) ratio. On day 7, the T cells in the co‐culture were collected, and the proportion of CAR^+^ cells was measured by flow cytometry. CAR^+^ cells were counted and replated in a new 6‐well plate at the same E:T ratio for the next round of co‐culture. This subculturing was repeated for at most six rounds.

The cytotoxic activities of T cells were evaluated using an xCELLigence realtime cell analyzer (RTCA) system (ACEA Biosciences, San Diego, California, USA). For the assay, 1 × 10^5^ tumor cells per well were seeded into 16‐well microtiter plates for 24 h to form a monolayer. Next, different E:T ratios of CAR‐T cells were added to the plate, and the cell index–time plot was recorded.

### Detection of Anti‐PD‐1 Nanobody

A quantitative electrochemiluminescence (ECL) method was designed to monitor the PD‐1 nanobody concentration. The method was used for mouse and human peripheral blood samples as well as homogenized tissue samples from a mouse xenograft tumor. A customized MULTI‐ARRAY 96 Sm Spot plate (Mesoscale Discovery, MSD, L45XA‐3) was coated with PD‐1 antigen (5 µg mL^−1^) overnight, then washed with phosphate‐buffered saline with Tween 20 (PBST) buffer and blocked with 3% bovine serum albumin (BSA) for 30 min. Assay controls and serum samples were diluted to 10% serum with assay buffer and added to a plate for 2 h. After incubation, washing with PBST, a SULFO‐TAG‐labeled antibody (0.75 µg mL^−1^) was added to the plate for another 2 h. Final addition of SULFO‐TAG streptavidin (0.25 µg mL^−1^) resulted in an ECL signal that was measured in relative light units (RLU) using the MSD QuickPlex SQ 120 plate reader.

To measure the antibody concentration in the tumor, snap‐frozen tumor tissue was weighed and homogenized in T‐PER tissue protein extraction reagent with protease inhibitors (Roche) as lysis buffer. Every 60 mg of tumor tissue was homogenized in 400 µL lysis buffer, processed by tissue homogenizer (TIANGEN). The whole process was performed on ice. The homogenized tissue lysate was placed in a pre‐cooled centrifuge at 4 °C for 12 000 g and centrifuged for 15 min. The supernatant was carefully collected as tumor lysate, and the concentration of the PD‐1 nanobody was measured thereafter.

### Mouse Experiments

For anti‐tumor treatment, animal experiment protocols were approved by Joinn Laboratories Co, Ltd (Suzhou, China, ethical approval #S‐ACU22‐0042) or Phenotek Biotechnology Inc. (Shanghai, China, ethical approval #AUP‐220221‐S01). Procedures were following the institutional guidelines for the care and use of animals. Six to eight‐week‐old female NOD‐Prkdc^scid^ IL2R^null^ (NOG) mice (Beijing Vital River Laboratory Animal Technology Co, Ltd) were injected with 1 × 10^7^ NCI‐H226 tumor cells that overexpressed PD‐L1 subcutaneously. When the tumors grew to a pre‐defined volume, mice were randomly divided into different groups and received infusions. For the mock‐T group, the total number of T cells was the same as that in the NAC‐T groups at the highest dose level. Tumor volume and body weight were monitored twice a week. Tumor volume (mm^3^) was calculated as follows: 1/2 × long diameter × (short diameter)^2^.

### Antigen Spreading

Antigen spreading of T‐cells was measured using a 96‐well IFN‐γ enzyme‐linked immunospot (ELISpot) assay plate (MabTech, Cat#3420‐2AST‐10). Patient PBMCs were plated at 3 × 10^5^ cells per well without any peptide as a control or with 15‐mer PepMix peptide pools of each indicated TAA (JPT Peptide Technologies, Germany) at 200 ng/pepmix/well for 24 h. IFN‐γ spots forming cells (SFC) were counted using an AID Elispot plate reader (AID vSpot, Germany). SFCs for each TAA were calculated via the actual spot count minus the control spot count.

### Immunogenicity Assay

ADA against either the MSLN CAR nanobody or the anti‐PD‐1 nanobody in patient serum was detected using a bridging ELISA. In brief, the MSLN CAR and anti‐PD‐1 nanobodies were labeled with biotin or Ru. MSD streptavidin plates were pre‐coated with biotin‐labeled target protein. Diluted samples were then added to the plate. The captured ADAs were then detected via the Ru‐labeled target protein, which generated an electrochemiluminescence signal in buffer containing tripropylamine.

### Statistical Analysis

All statistics were performed using GraphPad Prism v8.3.0. For comparison between two groups, data distribution was first tested, and an unpaired *t*‐test or Mann–Whitney U test was applied accordingly. One‐way ANOVA was used for comparisons among three or more groups. Two‐way ANOVA with mixed‐effects model was used for comparisons between groups in time‐related growth curves, and post hoc Turkey's multiple comparisons were employed to analyze data from the same time point.

## Conflict of Interest

Y.S., J.L., Y.X., Z.L., D.S., J.L., T.L., J.G., W.X., W.Z., Y.L., L.R., and Q.Q. are employees of the Shanghai Cell Therapy Group, Co, Ltd. Y.S., J.L., T.L., W.Z., and Q.Q. are inventors on patents related to the anti‐MSLN nanobody, the anti‐PD‐1 nanobody and the CIFT promoter. All other authors declare no competing interests. Shanghai Cell Therapy Group Co, Ltd. provided the investigate drugs (NAC‐T cells) and financial support for the clinical trial, such as patient compensation and examination costs. All clinical data have been reviewed and verified by the principal investigators independently.

## Author Contributions

Y.S., H.Y., Q.X., X.L., J.L., J.D., and J.X. contributed equally to this work. Q.Q., C.X., J.W., W.X., W.Z., and Y.L. supervised the study. Q.Q., Y.S., C.X., J.W., J.D., and L.R. conceptualized the study. J.W., Q.X., X.L., and J.L. were principal investigators of the clinical trial. Z.L. designed the clinical trial, wrote treatment protocols, and analyzed clinical data. J.D., Z.L., L.L., and Y.X. participated in patient treatments and analyzed CT images. J.L. designed the strategy and screened out the nanobodies. D.S. developed NAC‐T manufactory protocols and prepared NAC‐T cells. T.L. designed and prepared *piggyBac* mRNA and CAR/PD‐1 constructs. L.R. and J.G. designed and performed the in vitro and in vivo functional analyses. H.Y. performed bioinformatic analyses on patient samples. Y.S., H.Y., J.X., and L.R. wrote the manuscript. B.S., J.Z., J.W., C.X., and Q.Q. revised the manuscript.

## Supporting information



Supporting Information

## Data Availability

The data that support the findings of this study are available from the corresponding author upon reasonable request.
