# Supplementary material for: Anti‐PD‐1 Nanobody‐Armored MSLN CAR‐T Therapy for Malignant Mesothelioma: Preclinical and Clinical Studies
Source: Adv Sci (Weinh). 2025 Oct 24;13(35):e08754. doi: 10.1002/advs.202508754 (PMC13292173; doi:10.1002/advs.202508754)
Supplement: Supplementary file 1 — Supporting Information [file ADVS-13-e08754-s001.docx]

**Supporting Information**

**Anti-PD-1 Nanobody-armored MSLN CAR-T Therapy for Malignant Mesothelioma: Preclinical and Clinical Studies**

*Yan Sun^#^, Haochen Yang^#^, Qing Xu^#^, Xingya Li^#^, Jinxing Lou^#^, Jianchun Duan^#^, Jiachen Xu^#^, Zhuqing Liu, Yong Xia, Zhicai Lin**, Linlin Li, Dan Sun, Jiaguo Li, Tao Liu, Jun Guo, Wenfeng Xu, Weimin Zhu, Yi Liu, Boyang Sun, Jia Zhong, Lijie Rong^*^, Qijun Qian^*^, Chenqi Xu^*^, and Jie Wang^*^*

**Supporting Methods**

Patient eligibility

Key eligibility criteria included: (1) Age between 18 and 70 years; (2) Histological or cytological diagnosis of solid tumors, such as malignant mesothelioma; (3) Failure of available standard medical anti-cancer therapies; (4) Mesothelin expression of cells in tumor tissue greater than 50%, PD-L1 expression must be positive; (5) According to RECIST 1.1 criteria (for MPM, mRECIST applies), at least one evaluable or measurable lesion; (6) Life expectancy of more than 3 months; (7) Eastern Cooperative Oncology Group (ECOG) performance status score of 0 to 1.

Key exclusion criteria included: (1) Prior targeted therapy, gene therapy, or cell therapy against mesothelin (including CAR-T cell therapy); (2) Medical condition that requires chronic systemic steroid therapy or immunosuppressive medication; (3) Detectable clinically relevant central nervous system (CNS) metastasis and/or other diseases affecting CNS.

Flow cytometry

In preclinical and clinical studies, flow cytometry was used to measure the number of human T cells in peripheral blood and tumor tissue. The expression of CAR on CAR-T cells was examined using biotinylated human MSLN protein (synthesized by GenScript) followed by PE-Streptavidin conjugate (554061, BD Biosciences). Other antibodies (all anti-human) and reagents used for analysis included: AF700 CD3 antibody (300424, Biolegend), AF488 CD4 antibody (344618, Biolegend), and PerCP-Cy5.5 CD8a antibody (clone RPA-T8, 45-0088-42, eBioscience), BV605 CD45RO (304238, Biolegend), PE-Cy7 CCR7 (353226, Biolegend), APC CD95 (17-0959-42, eBioscience), PerCP Cy5.5 CD45 (304028, Biolegend), BV510 CD3 (564713, BD Biosciences), APC-Cy7 CD8 (557834, BD Biosciences), APC CD45RA (550855, BD Biosciences), AF488 CD4 (557695, BD Biosciences), BV421 CCR7 (353208, Biolegend), BB700 TIM3 (746178 BD Biosciences), AF647 LAG3 (565716, BD Biosciences), R718 TCF-1 (567587, BD Biosciences), Live/Dead fixable aqua fluorescent reactive dye (L34966, Invitrogen), Optilyse C lysing solution (A11895, BECKMAN COULTER), and BD Trucount Tubes (340334, BD Biosciences). To evaluate the receptor occupancy on T cells in tumor tissue, single-cell suspension of tumor tissues was prepared and stained using either of the following PD-1 antibodies: CD279-BV421 (Biolegend, clone #NAT105, Cat#367422) to detect free PD-1 molecules, and CD279-BV421(BD, clone #MIH4, Cat#564323) to detect all expressed PD-1 molecules. Data were collected on a CytoFLEX S (BECKMAN COULTER) and analyzed via Kaluza analysis.

Quantitative PCR

Patient blood samples were collected using EDTA tubes before and at different time points after infusion. Genomic DNA was extracted using the MagMAX DNA Multi-Sample Ultra Kit (Thermo Fisher Scientific, Cat#A36570). The copy number of the CAR gene in each sample was measured by quantitative PCR. Primer sequences were:

Forward: AGGGCCGCTTCACCATCAG；

Reverse: CCAGTCGGGGTCGTAGTCGTT；

Probe: TACATGCCGGTGTCCTCGGGCTT.

The standard curve ranging from 50 to 1 × 10^6^ copies was established using the CAR plasmid. The results were reported as copies of the CAR transgene per μg genomic DNA.

Immunohistochemistry

Immunohistochemistry analysis was used for screening of patients or validation of mouse xenograft tumors using formalin-fixed, paraffin-embedded tissue sections. In brief, the slides were deparaffinized in xylene and rehydrated in a graded alcohol series, and 3% hydrogen peroxide was used for elimination of endogenous peroxidase. Antigen retrieval was conducted in EDTA buffer (pH 9.0). After blocking non-specific binding with 3% goat serum in PBS, anti-human MSLN antibody (Cell Signaling Technology, clone D9R5G, Cat#99966), PD-L1 (Abcam, clone 73-10, Cat#ab228415), calretinin (Abcam, clone EP1798, Cat#ab92341), BAP-1 (Abcam, clone EPR22826-65, Cat#25561), WT-1 (Abcam, clone SP321, Cat#ab224806) and CK5/CK6 (Abcam, clone D5/16B4, Cat#ab17133) were used for staining. The process was performed on an automated immunostainer (Leica Bond-III, Dako Autostainer Link 48) using a Bond Polymer Refine Detection system.

Cytokine production

The concentration of cytokines was analyzed using a human V-PLEX Proinflammatory Panel 1 kit (Cat#K15049D-2, MSD) for IFN-γ, IL-1β, IL-2, IL-4, IL-6, IL-8, IL-10, IL-12p70, IL-13, and TNF-α. Experiments were performed according to the manufacturer’s instructions. All assays were performed in duplicate for each sample. The ECL signal was detected using MSD QuickPlex SQ 120 plate reader.

ADA blocking assay

To confirm the potential blocking effects of ADA against anti-PD-1 nanobody, we have established an ELISA-based assay to assess the binding of PD-1 to PD-L1, the blockade of the anti-PD-1 nanobody, and whether the ADA against nanobody impair such blockade. In brief, pre-coated human PD-1 bind to biotinylated PD-L1, and being detected using SA-HRP. The anti-PD-1 nanobody is added to this system to mask the PD-1 thus impair its binding to PD-L1. When the anti-PD-1 nanobody is pre-mixed with patient serum containing neutralizing ADA, the PD-1 binding to PD-L1 will be restored. A mouse mAb against the anti-PD-1 nanobody was developed and served as a positive control. The nanobody was pre-incubate with the mouse mAb before adding to the assay system, leading to a significant and dose-dependent impairment of PD-1 binding.

Integration site analysis

This experiment was conducted by Waker Bioscience Inc. (Shanghai, China). Polyclonal-Monoclonal Distance (PMD) analysis was performed using a method based on Rényi entropy (Afzal et al., 2019; Negre et al., 2015) with the ISOT software. Cell clonality was defined as an indicator of the rarity of integrated cells. The sample was considered polyclonal if no dominant cell clone was detected. If dominant cell clones were found, the sample was considered oligoclonal. The following two dimensions were used to assess cell diversity in each sample: the richness of the cells (number of independent integration sites) and the evenness of the cells (relative abundance of clones). The default clonality of a monoclonal cell sample was 0.

To make the relative clonal abundance of different cell richness comparable, an exponential curve was plotted for each sample, using data from our integration site analyses based on the following Rényi entropy formula:

$$H_{\alpha}= \frac{1}{1-\alpha}\log\sum_{i=1}^{S} p_{i}^{\alpha}$$

In this formula, α∈[0,+∞], S is the number of independent integration sites, and pi is the probability of integrated cell i. When α = 0, $H_{\alpha=0}$ = log *S*, which represents the richness of integration sites. When α approaches ∞, the Rényi entropy was $H_{\alpha\to\infty}=\log\frac{1}{\hat{p}}$ , where$\hat{p}$was the maximum proportional abundance of all integrated cells (the relative abundance of cells at independent integration sites). The abundance of cells with a top independent integration site decreases when the proportion of the cells is high, and vice versa.

In ISOT, the distribution bias of the integration site in each functional region of the host genome was analyzed by the position of the insertion site on the genomic DNA. The distribution of randomly inserted sites was used as the control distribution, named as in silico. And the distribution preference of the insertion sites in the chromosomes, the distribution preference of each chromosome, and the distribution preference of the insertion sites in the functional regions of each gene (such as the upstream and downstream 10kb region of the gene, exon, intron region, etc.) of the vector were compared and analyzed.

Tissue cross-reactivity assay

To assess the cross reaction of CAR and PD-1 nanobodies with antigens in non-malignant human tissues, cross reactivity was analyzed across 35 human tissues including the adrenal gland, aorta, bladder, blood cell, bone marrow, mammary gland, cerebellum, cerebrum, colon, duodenum, eye, esophagus, fallopian tube, stomach, heart, kidney, liver, lung, lymph nodes, ovary, pancreas, parathyroid, pituitary, placenta, prostate, skin, spinal cord, spleen, skeletal muscle, testis, thymus, thyroid, ureter, uterus cervix and uterus endometrium. Tissues were obtained from three independent donors. Frozen sections were subjected to immunohistochemistry staining using CAR or PD-1 nanobody conjugated with biotin (20.0 μg/mL), then antigen binding was detected using streptavidin-HRP. A human IgG1, kappa isotype antibody (MA14OC2903, Sino Biological) was labeled with biotin as negative control.

Surface plasmon resonance (SPR)

The interaction between antibody with antigen was monitored by SPR using a Biacore T200 (GE Healthcare) carried out in Multicycle mode with Sensor Chip Protein A (Cytiva). All the measurements were performed in buffer consisting of HBS-EP [10mM HEPES, 150 mM NaCl, 3mM EDTA, pH 7.4, and 0.05% (v/v) P20]. The antibody protein was captured on the chip at 50 to 100 response units. Then, gradient concentrations of protein (from 500 to 100 nM with five-fold dilutions) flowed over the chip surface and the real-time response was recorded. After each cycle, the sensor was regenerated with 10mM Gly-HCl (pH = 2.0). The raw data and affinities were collected and calculated using a 1:1 fitting model with BIA evaluation software (GE Healthcare, Biacore T200 Control Software and Biacore T200 Evaluation Software 3.1).

Evaluation of CAR tonic signaling

Using same strategy as a previously established experiment system (Chen et al., 2023), we evaluate the tonic signaling of MSLN CAR (VHH) versus CD19 CAR (FMC63 scFv) under the *piggyBac* non-viral transduction system and conventional lentiviral-based transduction system. In brief, the CAR construct was transduced into PBMC from a healthy donor, and cultured without stimulation of the target antigen. As a readout of CAR tonic signaling, the up-regulation of CD69 was measured by flow cytometry and the fold change of mean fluorescence intensity was normalized to the negative control (NC), which was T cells transduced with non-CAR lentivirus.

T cell receptor sequencing

PBMCs were isolated from patient before and after the CAR-T treatment by density gradient centrifugation using SepMate-50 (Cat#85460, Stemcell) and cryopreserved with CS10 freezing medium (Cat#100-1061, Stemcell) until use. Total DNA was isolated and CDR3 in the TCR β chain was amplified by multiplex PCR. Paired-end high-throughput sequencing of CDR3 was performed with a read length of 150 bp using Illumina NovaSeq 6000 platform.

Data cleaning was conducted using fastp (Beatty et al., 2018). Prior to assembly, overlapping read pairs were merged using FLASH (v.1.2.11). The MiXCR (v.3.0.4) software was used to map reads to the CDR3 sequence and assemble TCR sequences, and VDJtools (v1.2.1) was used for clone sequence analysis. The immune repertoire was characterized by evaluating the diversity and clonality. The diversity of the TCR repertoire was calculated based on the Shannon index (Yang et al., 2021), which represents both the relative number of clonotypes and the relative abundance or distribution of each clonotype, Shannon index=$-\sum_{i=1}^{s} \frac{ni}{N}\ln\frac{ni}{N}$. Clonality scores being defined as 1-(entropy)/log2 (number of productive unique sequences), indicated the diversity of clones and frequency of any given clone (Wang et al., 2020).

Single-cell sorting, library preparation, and sequencing

After frozen PBMCs were thawed, the number of survived cells was counted, which was generally above 80%. The cells that passed the test were washed and resuspended to prepare a suitable cell concentration of 700 to 1200 cells/μl. Then the cells were loaded approximately 18,000 cells per chip position using the 10× Chromium Next GEM Single Cell 5’ Kit v2. The system was operated on the machine. Gel bead in emulsions (GEMs) were constructed for single cell separation according to the number of cells to be harvested.

After GEMs had formed, they were collected for reverse transcription in a PCR machine for labeling. All subsequent steps were performed following the manufacturer’s protocols. GEMs were oil-treated, and the amplified cDNA was purified by magnetic beads, and then subjected to cDNA amplification and quality inspection. The 5’ gene expression library was constructed with the qualified cDNA. Purified libraries were sequenced on the Illumina NovaSeq 6000 Instrument using 150-bp paired-end reads.

Processing of scRNA-seq and scTCR-seq data and quality control

Cell Ranger and Cell Ranger V(D)J (version 7.0.1) were applied to filter low quality reads, align reads to the human reference genome (GRCh38), assign cell barcodes, and generate UMI matrices. The output gene expression matrices were analyzed using the R software (v4.3.2) with Seurat (version 4.3.1). All samples were merged into one Seurat object using the *merge* function in Seurat. Low quality cells with < 200 or >7000 genes detected, < 500 or >100,000 UMI counts detected or > 5% mitochondrial UMI counts detected were removed. All information of scTCR-seq were merged into Seurat object by *AddMetaData* function.

Dimension reduction, unsupervised clustering and cell type annotation

Dimension reduction and unsupervised clustering were performed according to the standard workflow in *Seurat*. *SCTransform* function was applied to normalize and find highly variable genes (HVGs) within the single-cell gene expression data. Mitochondrial genes, dissociation-induced genes and HLA genes were removed from HVGs for downstream analyses. Then, the effect of the percentage of mitochondrial gene counts was regressed out by using *SCTransform* function with parameter “vars.to.regress = ‘percent.mt’”. A principal component analysis (PCA) matrix was calculated to reduce noise by using *RunPCA* with default parameters. After PCA analysis, we use *Findneighbors* and *FindClusters* (by Louvain algorithm) function provided by Seurat. Then UMAP and graph-based clustering were performed on the object for visualization and cell clustering by *RunUMAP* function. The main immune cell types were annotated based on the expression pattern of DEGs and the well-known cellular markers from the literature. The number of principal components was 30 which following *Seurat* suggestion. DEGs were detected using the *FindAllMarkers* function with default parameters. Finally, we got 16 clusters for further analysis under parameter *resolution = 0.6* condition.

TCR diversity and expansion analysis of scTCR-seq

The processed scTCR-seq data were loaded using R package *scRepertoire* (version 2.0.0) function *combineTCR*. Then use function *quantContig* and *compareClonotypes* to annotate the TCR clone and find shared TCR clone by different samples. Use function *clonalDiversity* to calculate the clone diversity score. Moreover, we added the TCR clone expansion information onto *Seurat* object and use function *Dimplot* to display the TCR expansion level between different cell cluster and different samples.

**References for Supporting Methods**

Chen, J., Qiu, S., Li, W., Wang, K., Zhang, Y., Yang, H., Liu, B., Li, G., Li, L., Chen, M., et al. (2023). Tuning charge density of chimeric antigen receptor optimizes tonic signaling and CAR-T cell fitness. Cell Res 33, 341-354.

Afzal, S., Gil-Farina, I., Gabriel, R., Ahmad, S., von Kalle, C., Schmidt, M., and Fronza, R. (2019). Systematic comparative study of computational methods for T-cell receptor sequencing data analysis. Brief Bioinform 20, 222-234.

Beatty, G. L., O’Hara, M. H., Lacey, S. F., Torigian, D. A., Nazimuddin, F., Chen, F., Kulikovskaya, I. M., Soulen, M. C., McGarvey, M., Nelson, A. M., et al. (2018). Activity of Mesothelin-Specific Chimeric Antigen Receptor T Cells Against Pancreatic Carcinoma Metastases in a Phase 1 Trial. Gastroenterology 155, 29-32.

Negre, O., Bartholomae, C., Beuzard, Y., Cavazzana, M., Christiansen, L., Courne, C., Deichmann, A., Denaro, M., de Dreuzy, E., Finer, M., et al. (2015). Preclinical evaluation of efficacy and safety of an improved lentiviral vector for the treatment of beta-thalassemia and sickle cell disease. Curr Gene Ther 15, 64-81.

Wang, F., Xie, X., Song, M., Ji, L., Liu, M., Li, P., Guan, Y., Lin, X., Qin, Y., Xie, Z., et al. (2020). Tumor immune microenvironment and mutational analysis of tracheal adenoid cystic carcinoma. Ann Transl Med 8, 750.

Yang, H., Wang, Y., Jia, Z., Wang, Y., Yang, X., Wu, P., Song, Y., Xu, H., Gu, D., Chen, R., et al. (2021). Characteristics of T-Cell Receptor Repertoire and Correlation With EGFR Mutations in All Stages of Lung Cancer. Front Oncol 11, 537735.

**Supporting Figures and legends**


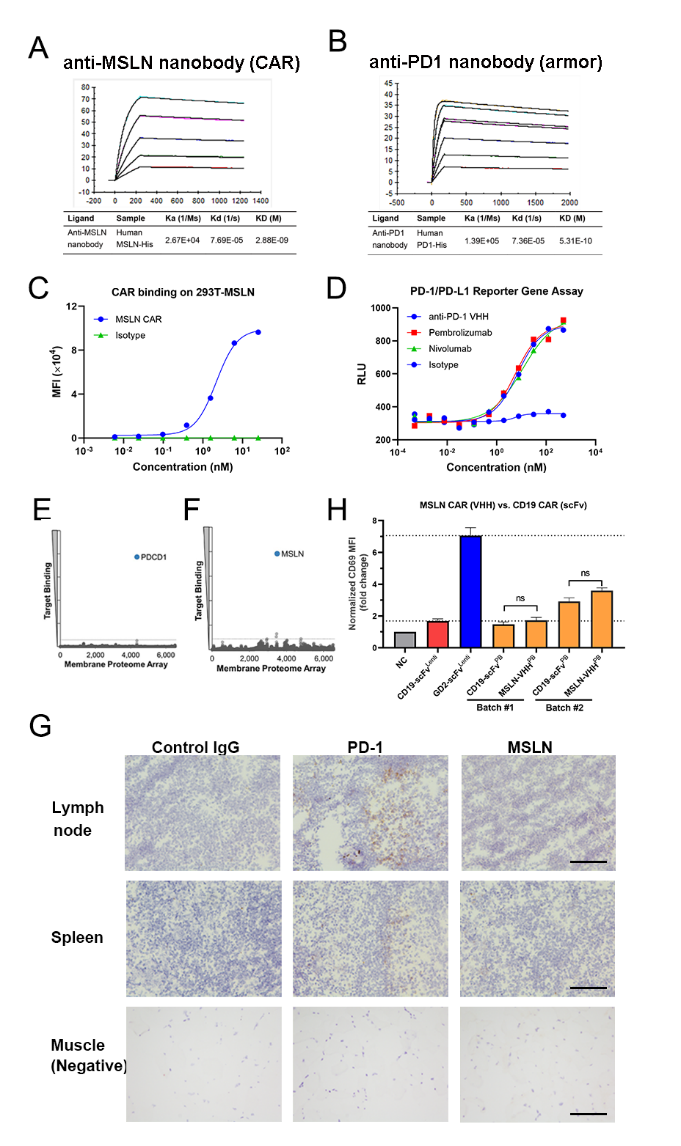


Figure S1. Antigen-binding specificity and functionality of the VHH nanobodies used in NAC-T.

**(A-F)** Characterization of anti-MSLN nanobody for the CAR structure and anti-PD-1 nanobody for the armor. Shown are the antigen binding affinity assessed by Surface Plasmon Resonance (SPR, A and B), the functional binding of the CAR nanobody to MSLN expressed on cell surface by flow cytometry (C), the functional blocking effect of the anti-PD-1 nanobody in comparison with pembrolizumab or nivolumab assessed by reporter gene assay (D), and the specificity of each nanobody assessed by Membrane Proteome Array (MPA, E and F).

MFI: Mean fluorescence intensity; RLU: Relative light units

**(G)** Results of tissue cross reaction assay of the anti-PD-1 and anti-MSLN CAR nanobodies across 35 human tissues. As expected, only weak to moderate binding activity of anti-PD-1 nanobody was detected on the immune cells in spleen and lymph node. Representative images are shown. Scale bar, 100 µm.

**(H)** Evaluation of CAR tonic signaling reflected by CD69 surface level of T cells transduced with anti-MSLN nanobody CAR or commercial anti-CD19 scFv CAR. The gene modification system was either lentiviral based (Lenti) or *piggyBac* transposon based (PB).


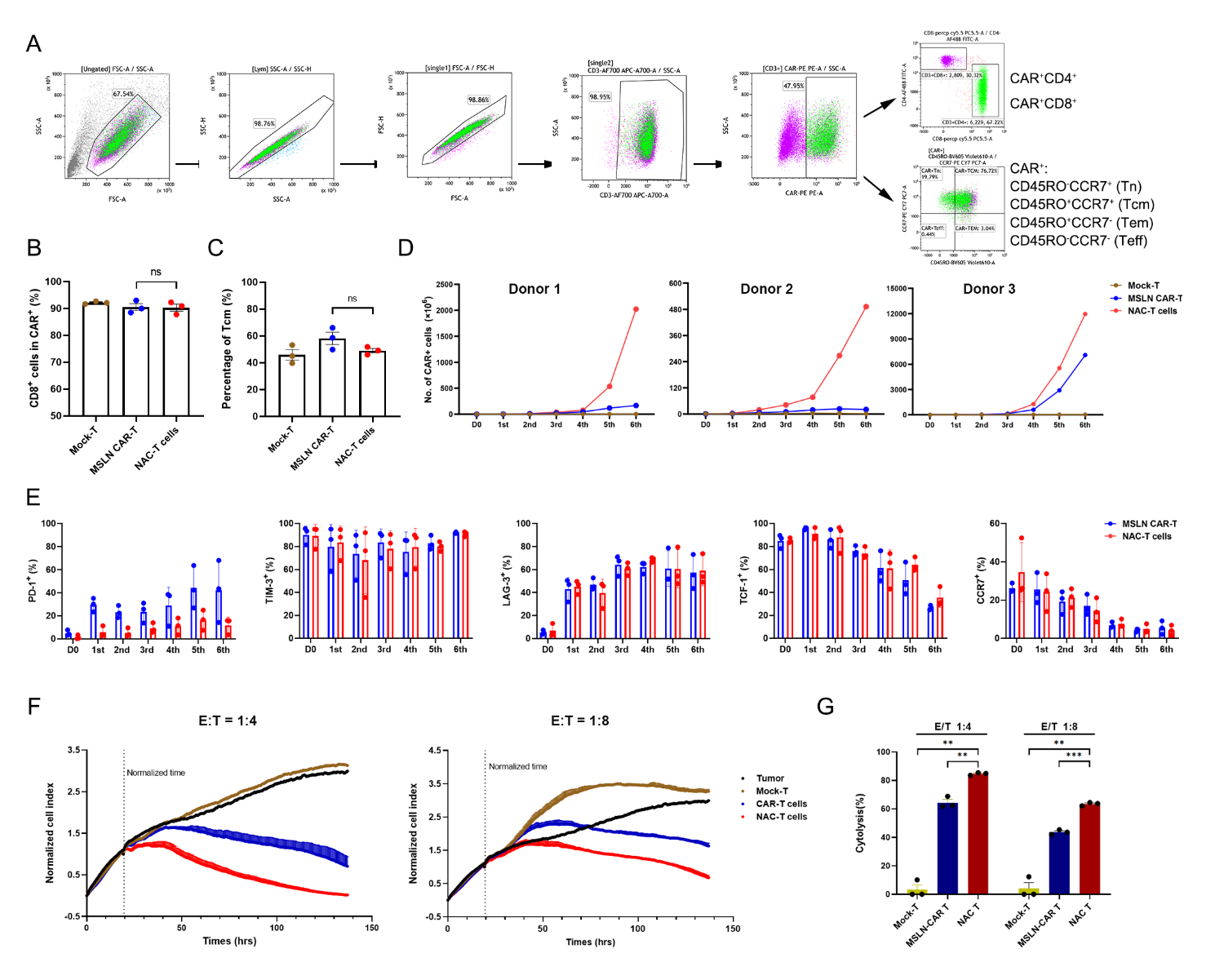


Figure S2. NAC-T characterization and its enhanced proliferation and anti-tumor activity *in vitro*.

**(A**–**C)** Basic characterizations of CAR-T subpopulations before cryopreservation. Mock-T, MSLN CAR-T and NAC-T cells have been cultured for 9 days post gene transduction, without antigen stimulation. **(A)** Flow cytometry gating strategy to analyses on NAC-T subtypes, shown as CD4^+^ versus CD8^+^ analyses (upper panel) and memory vs. effector T cell subpopulation analyses (lower panel). Data shown are **(B)** CD8^+^ proportion and **(C)** central memory **(**Tcm) proportion in NAC-T cells derived from three independent healthy donors.

(**D-E**) Enhanced proliferation of NAC-T during serial stimulation with NCI-H226 tumor cells. (**E**) Each figure shows the proliferation of NAC-T and MSLN CAR-T cells from the same donor. (**F**) Expression of the exhaustion-related markers including PD-1, TIM-3, LAG-3, TCF-1 and CCR7 in each round of serial stimulation.

**(F and G)** Improved anti-tumor activity of NAC-T when co-cultured with NCI-H226 tumor cells in RTCA killing assay. The effector-to-tumor (E:T) ratios were 1:4 and 1:8. Shown are **(F)** tumor killing curves and **(G)** corresponding cytolysis rates after 96 hours of co-culturing.

The data are shown as mean ± SEM from triplicates. ***p* < 0.01; ****p* < 0.001; ns, not significant by one-way ANOVA with multiple comparisons.


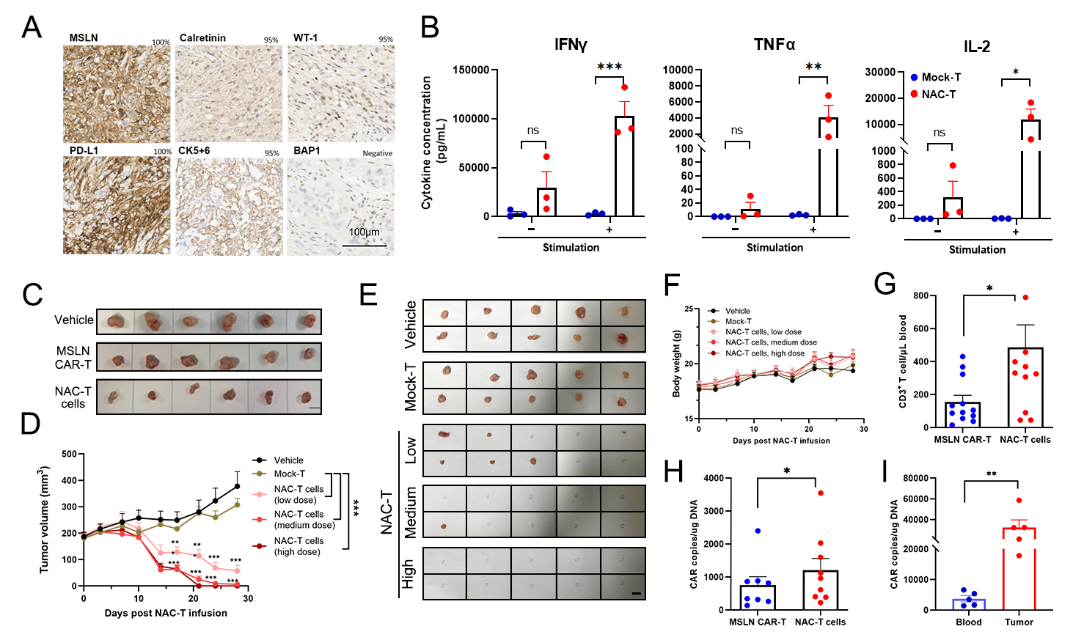


Figure S3. Dose-dependent anti-tumor activity of NAC-T cells in tumor-bearing mice.

**(A)** Characterization of mouse xenograft tumor established from NCI-H226 cells. IHC staining showing expression of mesothelioma markers such as calretinin, WT1 and CK5+6, as well as the lack of BAP1 expression in tumor cells but not surrounding stromal cells. Scale bar, 100 µm.

**(B)** NAC-T producing activation related cytokines after NCI-H226 cell stimulation by co-culturing. Shown are data from three independent donors.

**(C)** Images for all tumors related to Figure 2D. Scale bar, 1cm.

**(D-E)** Tumor growth curve (D) and all tumor images (E) show changes of tumor volume after NCI-H226 tumor bearing mice were infused with vehicle, mock-T or indicated doses of NAC-T cells. Data were collected from 10 mice per group. Low dose: 0.5 × 10^6^; medium dose: 1.5 × 10^6^; high dose: 5.0 × 10^6^ CAR^+^ cells per mouse. Scale bar, 1cm.

**(F)** Changes of mouse weight in (D) show limited toxic effect after NAC-T treatment.

**(G and H)** Promoted expansion of NAC-T compared with MSLN CAR-T. **(G)** Increased number of human CD3^+^ T cells and **(H)** CAR copy number detected in mouse peripheral blood.

**(I)** Copies of CAR transgene in peripheral blood (blue) and tumor tissue (red) in tumor bearing mice treated with NAC-T.

Data are shown as mean ± SEM. **p* < 0.05; ***p* < 0.01; ns, not significant. ****p* < 0.001 by two-way ANOVA with multiple comparisons (B and D) or Mann-Whitey U test (G, H and I).


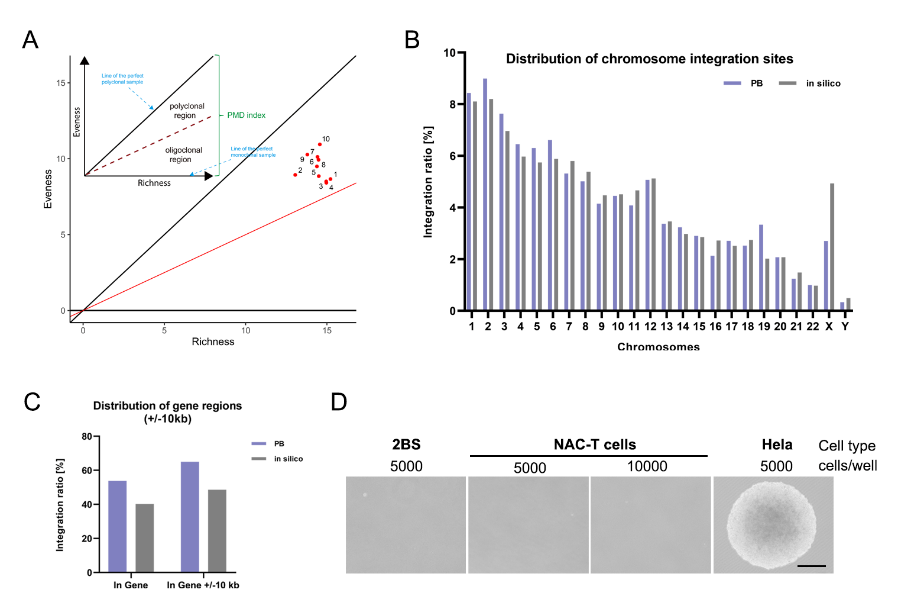


Figure S4. Assessment of safety profile of NAC-T cells *in vitro*.

**(A**–**C)** Analyses of gene integration sites by LTA-PCR in ten NAC-T samples derived from independent donors. **(A)** The cloning plane showing the polyclonal monoclonal distance (PMD) index of each NAC-T sample. All of the 10 samples (red dots) were mapped in the polyclonal region. The location of integration sites **(B)** across chromosomes and **(C)** in gene coding regions were also analyzed. Gray bars represent a predicted pattern of random distribution. Blue bars indicate the detected proportion of integration sites in NAC-T samples.

**(D)** The tumorigenic potential of NAC-T cells was evaluated using soft agar clone formation assay. Human cervical carcinoma cell line Hela served as positive control. Human fetal lung fibroblasts cell line 2BS served as negative control. A total of 5000 or 10000 NAC-T cells were tested. Representative images from triplicate wells are shown. Scale bar, 200 µm.


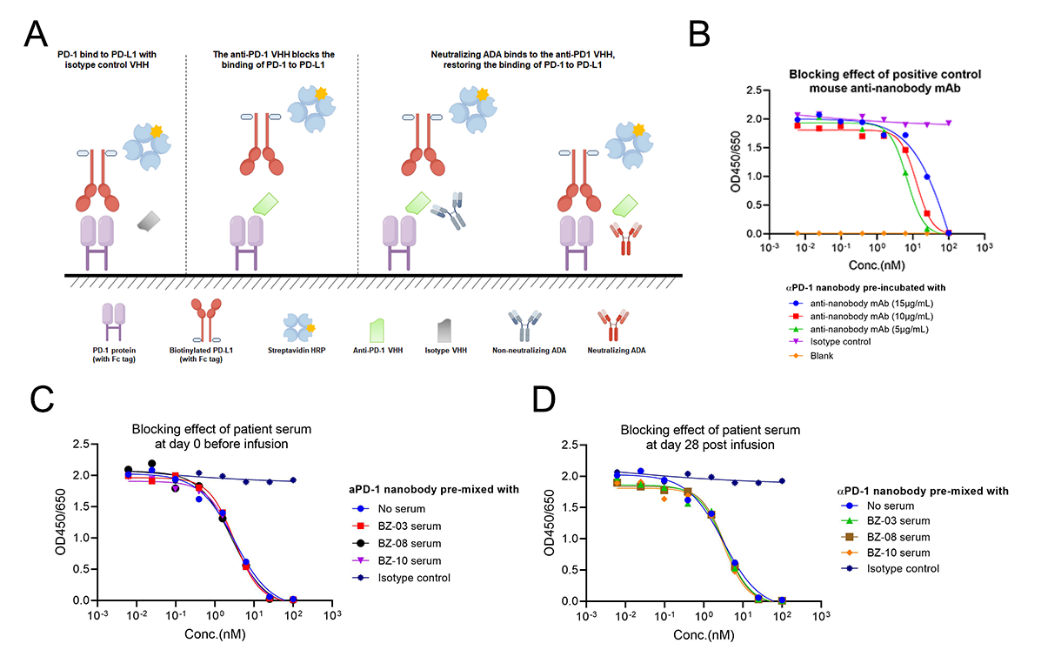


Figure S5. ADA in patient samples show limited blocking effect on PD-1 binding to PD-L1.

**(A)** Schematic illustration of the ELISA based PD-1 blocking assay. In brief, pre-coated human PD-1 bind to biotinylated PD-L1, and being detected using SA-HRP. The anti-PD-1(αPD-1) nanobody is added to this system to mask the PD-1 thus impair its binding to PD-L1. When the αPD-1 nanobody is pre-mixed with patient serum containing blocking ADA, the PD-1 binding to PD-L1 will be restored.

**(B)** Assay validation using a mouse mAb against αPD-1 nanobody, served as positive control. The αPD-1 nanobody was pre-incubate with the mouse mAb before adding to the assay system. A significant and dose-dependent impairment of PD-1 binding was observed.

**(C-D)** The blocking effect was evaluated using the αPD-1 nanobody pre-mixed with serum patient serum collected at day 0 (**C**) and day 28 (**D**) post the last NAC-T infusion. The ADA in patient serum was test negative (BZ-03), positive with low titer (BZ-08) or high titer (BZ-10).


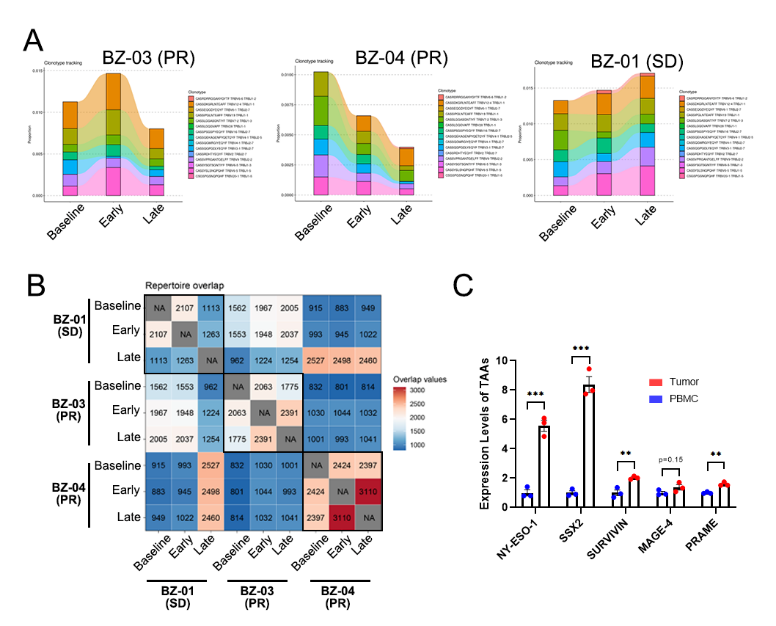


Figure S6. TCR and single cell sequencing data and clone expansion analyses.

**(A)** Sankey diagram tracking the Top 15 TRB clones that have highest clonotype frequency in three patients among three time points. Colors represent different clonotype and bar heights reflect clonotype proportion.

**(B)** Heatmap showing TCR repertoire overlap values across all defined cell clusters and divided by patients. Higher value reflects more repertoire overlap level.

(**C**) Expression level of indicated TAAs in tumor biopsy vs. PBMC from patient BZ-03 measured by qPCR. Bar indicates mean ± SEM from 3 technical replicates per TAA. ***p*<0.01; ****p*<0.001 by multiple unpaired t-test.


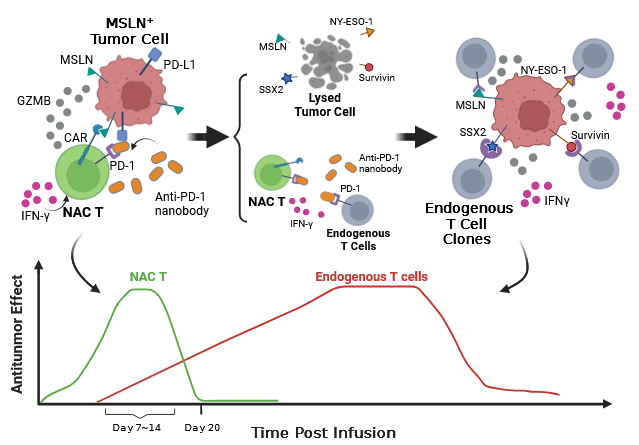


1)

2)

3)

**Figure S7. Illustration of NAC-T dual-stage mechanism-of-action.**
1) Primary anti-tumor activity of NAC-T mediated by the MSLN CAR binding. Intratumoral NAC-T cells are activated by MSLN-expressing tumor cells and directly lyse them. The activation of NAC-T cells induces the secretion of anti-PD-1 nanobody to block surface PD-1. In autocrine manners, the self-secreted nanobodies prevent NAC-T cells from exhaustion and lead to improved T-cell expansion; 2) Modification of tumor microenvironment to break immunosuppression. The lysed tumor cells release tumor antigens, including MSLN and other TAAs such as SSX2, NY-ESO-1 and survivin into the tumor microenvironment. In paracrine manners, the activated NAC-T cells secrete anti-PD-1 nanobody to impair the exhaustion of bystander tumor-infiltrated T cells and produce inflammatory cytokines that improve anti-tumor immune responses. 3) Secondary anti-tumor activity mediated by the TAA-responsive bystander T cells. The newly released TAAs induce a subsequent activation and expansion of surrounding T cells to coordinate with NAC-T to lyse tumor cells in the microenvironment.

Supplementary Tables

Table S1. Location of top 10 common integration sites (CIS) in NAC-T samples.

| Top10 CIS | Chr | Locus | Dimension | Gene name |
| --- | --- | --- | --- | --- |
| Top 1 | 3 | 18400545 | 471346 | SATB1 SATB1-AS1 KCNH8 |
| Top 2 | 6 | 89910436 | 432900 | GJA10 BACH2 MIR4464 |
| Top 3 | 14 | 61275572 | 324472 | TMEM30B PRKCH LOC101927780 FLJ22447 |
| Top 4 | 3 | 105595132 | 934460 | ALCAM CBLB LINC00882 |
| Top 5 | 3 | 70512302 | 1153832 | FOXP1 EIF4E3 |
| Top 6 | 11 | 127874144 | 793071 | LOC101929497 ETS1 FL11 |
| Top 7 | 7 | 110295840 | 1369780 | EIF3IP1 IMMP2L DOCK4 |
| Top 8 | 5 | 35385484 | 496039 | PRLR SPEF2 IL7R |
| Top 9 | 5 | 39040455 | 281284 | RICTOR FYB C9 |
| Top 10 | 11 | 14611579 | 298764 | PSMA1 PDE3B CYP2R1 |

Table S2. Characterization of NAC-T cell products used in clinic trial.

| **Patient**  **ID** | **Living cells (%)** | **CD3^+^ T**  **(%)** | **CAR^+^ in**  **CD3^+^ (%)** | **CD4^+^ in**  **CAR^+^CD3^+^ (%)** | **CD8^+^ in**  **CAR^+^CD3^+^ (%)** | **Cytotoxicity (%)** | **αPD-1 vector (copy/cell)** | **CAR vector (copy/cell)** |
| --- | --- | --- | --- | --- | --- | --- | --- | --- |
| **BZ-01** | 81.10 | 100.00 | 41.92 | 20.75 | 77.35 | 79.00 | 6.00 | 4.40 |
| **BZ-02** | 77.30 | 98.30 | 77.93 | 55.79 | 26.59 | 11.10 | 15.30 | 6.40 |
| **BZ-03** | 89.00 | 99.43 | 10.96 | 37.42 | 60.89 | 79.30 | 9.80 | 7.20 |
| **BZ-04** | 89.00 | 99.60 | 45.10 | 22.06 | 77.35 | 69.40 | 6.00 | 4.40 |
| **BZ-05** | 86.30 | 100.00 | 45.12 | 53.78 | 40.43 | 65.30 | 3.20 | 3.20 |
| **BZ-06** | 88.70 | 99.51 | 38.11 | 8.91 | 91.07 | 77.10 | 6.30 | 5.70 |
| **BZ-07** | 70.10 | 99.85 | 46.20 | 50.68 | 47.83 | 66.70 | 48.80 | 93.80* |
| **BZ-08** | 92.20 | 99.96 | 40.70 | 11.63 | 87.31 | 50.30 | 3.50 | 3.10 |
| **BZ-09** | 86.62 | 98.94 | 35.47 | 14.60 | 79.48 | 89.60 | 8.30 | 6.10 |
| **BZ-10** | 87.70 | 99.72 | 35.07 | 16.48 | 83.46 | 79.90 | 7.00 | 8.30 |
| **BZ-11** | 93.00 | 99.94 | 52.55 | 21.10 | 78.70 | 50.20 | 8.10 | 9.80 |
| **Median** | 87.70 | 99.72 | 41.92 | 21.10 | 77.35 | 69.40 | 7.00 | 6.10 |

* For this patient, NAC-T cells were manufactured with a 5-day fast protocol, in which a substantial proportion of electroporated plasmids have not been integrated into the genome. Such cytoplasmic vector contributes to the overall vector copy number as well as the integrated vectors, leading to a particularly high copy number being detected.

Table S3. Baseline characteristics and treatment responsiveness of patients.

| **Patient ID** | **Type of mesothelioma** | **Pathological subtype** | **Tumor stage** | **Prior anti-**  **PD-1/PD-L1** | **1^st^ infusion (10^7^/kg)** | **2^nd^ infusion (10^7^/kg)** | **Target expression** | | **Best response** |
| --- | --- | --- | --- | --- | --- | --- | --- | --- | --- |
|  |  |  |  |  |  |  | **MSLN (%)** | **PD-L1 (%)** |  |
| **BZ-01** | Pleural | Epithelioid | III | No | 1.5 | 1.5 | 100 | 25 | SD |
| **BZ-02** | Peritoneal | Biphasic | III | Yes | 0.5 | 0.75 | 100 | 6.7 | CR |
| **BZ-03** | Pleural | Epithelioid | IV | No | 1.0 | 1.0 | 100 | 70 | PR |
| **BZ-04** | Pleural | Epithelioid | IV | No | 1.5 | N/A | 100 | 1 | PR |
| **BZ-05** | Pleural | Epithelioid | IV | Yes | 1.5 | 1.5 | 100 | 75 | SD |
| **BZ-06** | Peritoneal | Epithelioid | III | No | 2.0 | N/A | 95 | 60 | PR |
| **BZ-07** | Pleural | Epithelioid | III | No | 0.5 | N/A | 100 | 90 | SD |
| **BZ-08** | Peritoneal | Epithelioid | II | No | 1.0 | N/A | 95 | 30 | SD |
| **BZ-09** | Peritoneal | Epithelioid | II | No | 2.0 | N/A | 90 | 30 | PR |
| **BZ-10** | Peritoneal | Epithelioid | III | No | 2.0 | N/A | 100 | 1 | PR |
| **BZ-11** | Peritoneal | Epithelioid | III | No | 2.0 | N/A | 100 | 40 | PR |

Table S4. Treatment related adverse events (TRAEs) over grade 3.

| **TRAEs**  **≥ Grade 3, N (%)** | **DL1: 5×10^6^**  **(N=2)** | | **DL1: 10×10^6^**  **(N=2)** | | **DL3: 15×10^6^**  **(N=3)** | | **DL4: 20×10^6^**  **(N=4)** | | **All dose levels**  **(N=11)** | |
| --- | --- | --- | --- | --- | --- | --- | --- | --- | --- | --- |
|  | **Any grade** | **≥ Grade 3** | **Any grade** | **≥ Grade 3** | **Any grade** | **≥ Grade 3** | **Any grade** | **≥ Grade 3** | **Any grade** | **≥ Grade 3** |
| Leukopenia | 0 | 0 | 1 (50) | 1 (50) | 3 (100) | 3 (100) | 4 (100) | 3 (75) | 8 (73) | 7 (64) |
| Neutropenia | 0 | 0 | 2 (100) | 0 | 3 (100) | 3 (100) | 2 (50) | 2 (50) | 7 (64) | 5 (45) |
| Lymphopenia | 1 (50) | 1 (50) | 0 | 0 | 3 (100) | 3 (100) | 2 (50) | 1 (25) | 6 (55) | 5 (45) |
| Anemia | 1 (50) | 0 | 2 (100) | 0 | 3 (100) | 2 (67) | 1 (25) | 0 | 7 (64) | 2 (18) |
| Thrombocytopenia | 0 | 0 | 1 (50) | 0 | 3 (100) | 1 (33) | 2 (50) | 1 (25) | 6 (66) | 2 (18) |
| Increased IL-6 | 2 (100) | 0 | 2 (100) | 0 | 2 (67) | 0 | 2 (50) | 1 (25) | 8 (73) | 1 (9) |
| Fever | 1 (50) | 0 | 2 (100) | 1 (50) | 3 (100) | 0 | 2 (50) | 0 | 8 (73) | 1 (9) |
| Pneumonitis | 0 | 0 | 1 (50) | 0 | 1 (33) | 0 | 1 (25) | 1 (25) | 3 (27) | 1 (9) |
| Pleuritic pain | 0 | 0 | 0 | 0 | 0 | 0 | 1 (25) | 1 (25) | 1 (9) | 1 (9) |

TRAEs include events that were definitely, probably and possibly related to CAR-T therapy.

Adverse events were graded according to CTCAE v5.0, CRS was graded according to ASTCT criteria. CRS, cytokine release syndrome.

Table S5. Immunogenicity of the CAR and the anti-PD-1 nanobody.

| **Titer of ADA against CAR** | | | | | | | | **Titer of ADA against CAR** | | | | | | | |
| --- | --- | --- | --- | --- | --- | --- | --- | --- | --- | --- | --- | --- | --- | --- | --- |
| **Patient ID** | **D0** | **D28** | **II-D28** | **M2** | **M3** | **M6** | **M9** | **D0** | **D28** | **II-D28** | **M2** | **M3** | **M6** | **M9** |  |
| **BZ-01** | 0 | 0 | 0 | - | 0 | - | - | 0 | 0 | 0 | - | 0 | - | - |  |
| **BZ-02** | 0 | 1 | 45.7 | - | 65 | 41.3 | 0 | 23.2 | 28.5 | 192 | - | 234 | 168 | 140 |  |
| **BZ-03** | 0 | - | 0 | - | - | - | - | 0 | - | 0 | - | - | - | - |  |
| **BZ-04** | 0 | 0 | 69.8 | 0 | - | - | - | 0 | 61.3 | 0 | 7.6 | - | - | - |  |
| **BZ-05** | 0 | 0 | 0 | - | - | - | - | 0 | 0 | 1 | - | - | - | - |  |
| **BZ-06** | 0 | 1 | 403 | 2.91 | 6.5 | 15.8 | - | 2.55 | 3.43 | 2.88 | 3.92 | 2.98 | 3.63 | - |  |
| **BZ-07** | 0 | 2.88 | - | 1940 | - | - | - | 0 | 1.28 | - | 0 | - | - | - |  |
| **BZ-08** | 0 | 0 | - | 0 | - | - | 0 | 73.9 | 69.1 | - | 73.9 | - | - | 26.3 |  |
| **BZ-09** | 0 | 1 | - | 16.8 | - | 205 | 621 | 0 | 0 | - | 0 | - | 0 | 0 |  |
| **BZ-10** | 0 | 0 | - | - | 0 | - | - | 2000 | 3100 | - | - | 706 | - | - |  |
| **BZ-11** | 0 | 0 | - | - | - | 8.29 | - | 1 | 0 | - | - | - | 0 | - |  |

Note: II indicates the second infusion. D: days after infusion; M: month after the first infusion. Zero (0) means the sample was ADA negative. Dash (-) means sample was not available.
